# RAX2: a genome-wide detection method of condition-associated transcription variation

**DOI:** 10.1093/nar/gkv411

**Published:** 2015-05-07

**Authors:** Yuan-De Tan, Jixin Deng, Joel R Neilson

**Affiliations:** 1 Department of Molecular Physiology and Biophysics, Baylor College of Medicine, One Baylor Plaza, Houston, TX 77030, USA; 2 Dan L. Duncan Cancer Center, Baylor College of Medicine, One Baylor Plaza, Houston, TX 77030, USA

## Abstract

Most mammalian genes have mRNA variants due to alternative promoter usage, alternative splicing, and alternative cleavage and polyadenylation. Expression of alternative RNA isoforms has been found to be associated with tumorigenesis, proliferation and differentiation. Detection of condition-associated transcription variation requires association methods. Traditional association methods such as Pearson chi-square test and Fisher Exact test are single test methods and do not work on count data with replicates. Although the Cochran Mantel Haenszel (CMH) approach can handle replicated count data, our simulations showed that multiple CMH tests still had very low power. To identify condition-associated variation of transcription, we here proposed a ranking analysis of chi-squares (RAX2) for large-scale association analysis. RAX2 is a nonparametric method and has accurate and conservative estimation of FDR profile. Simulations demonstrated that RAX2 performs well in finding condition-associated transcription variants. We applied RAX2 to primary T-cell transcriptomic data and identified 1610 (16.3%) tags associated in transcription with immune stimulation at FDR < 0.05. Most of these tags also had differential expression. Analysis of two and three tags within genes revealed that under immune stimulation short RNA isoforms were preferably used.

## INTRODUCTION

**Figure 1.**
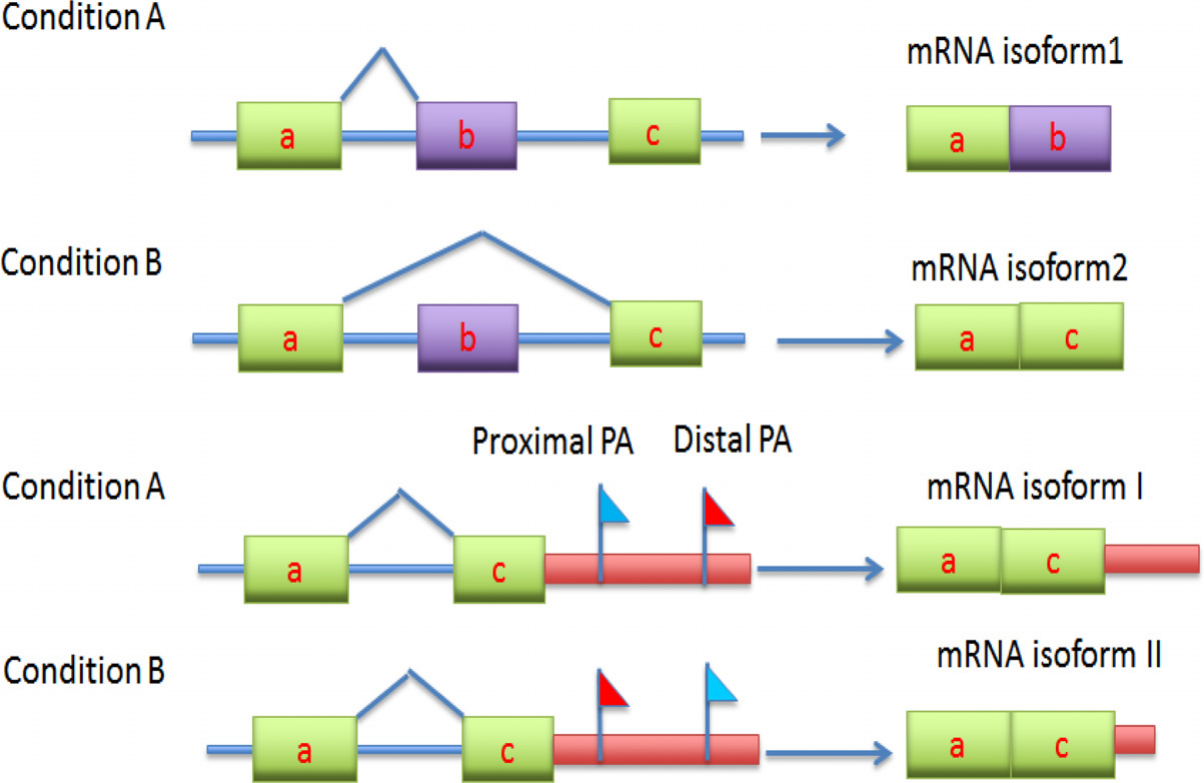
Demonstration for association of usage of alternative splice and poly(A) sites with conditions: splicing switch and usage switch of poly(A) sites due to change in condition. Under normal condition, the gene transcription product is isoform 1 due to splicing event between exons a and b, but in stress or stimulation, for example, the gene product is isoform 2 by switching splicing from exon b to exon c (top two figures). This is called a splicing switch. Likewise, under normal condition, cells use distal poly(A) site and the transcription produces mRNA isoform I but when cells are stressed or stimulated, poly(A) site usage switches from distal site to proximal site and produces isoform II (bottom two figures). This is called poly(A) site usage switch.

It has recently been revealed that alternative splicing and alternative cleavage and polyadenylation (ACP) is not only a universal post-transcription processing step in eukaryotic gene expression but also a versatile mechanism for post-transcriptional regulation of genes (1–3) . After transcription, a pre-mRNA is capped at the 5′ end, spliced and cleaved in the 3′-untranslated region (3′ UTR) to yield a new open end that allows to add a polyadenylation (poly(A)) tail (3,4). A poly(A) tail at 3′ end may protect the mRNA from unregulated degradation, trigger export of the mRNA to cytoplasm and assist recognition by translation machinery (3–5). A poly(A) signal at which the pre-mRNA is cleaved and referred to as poly(A) site is recognized and activated by a batch of protein factors called polyadenylation factors (3,4,6,7). Alternative splice and poly(A) sites significantly increase the complexity of transcriptomes and proteomes because they lead to multiple isoforms or variants of an mRNA. Using next-generation sequencing (NGS) techniques, it has been observed that over 50% of transcription units in the mammalian genome are characterized by ACP (8–13). As alternative splice and poly(A) sites can uncover most transcription variation at subgene level, to investigate association of transcription variation with tissue types or cell state change has become very important for exploring etiologic mechanism of disease of interest (8,14–20). But almost all of the current studies are based on differential analysis. This may be because the current large-scale statistical methods such as DESeq (21), baySeq (22), edgeR exact test (23,24), edgeR GLM (25), DEXSeq (26), Cuffdiff (27,28), DiffSplice (29), SplicingCompass (30) are differential analysis methods. Of these methods, the former four are used to identify differential transcription between conditions and the latter four focus on finding differential splicing between conditions. However, unlike gene differential expression, splice switch or usage switch of alternative poly(A) sites occurs on the same transcription unit and are associated with a change in condition (Figure 1) . As shown in Figure 1, switch usage or switch splicing is involved in two conditions and two poly(A) sites or two splice sites:

**Table:** 

	condition 1	condition 2
site A	high expression	low expression
site B	low expression	high expression

High-expression switches from site A in condition 1 to site B in condition 2. This is a case that high expression of sites is associated with change in condition. If one just looks at the difference in expression of site A or site B between two conditions, then this is another case that one concerns differential expression of single sites between two conditions. In the first case only an association method can be used to test if sites A and B are associated with conditions 1 and 2 in expression but for the second case one can use differential methods to test for differential expression of each site between two conditions. So the use of differential analysis methods to identify usage and splicing switches from site to site on the same RNA unit is not ideal. To our best knowledge, no existing large-scale association analysis methodologies have been proposed. The traditional association methods such as Pearson chi-square test and Fisher exact test are single-test methods and work on the count data without replicates, they hence could not directly be applied to high-throughput transcriptomic count data of RNA reads with replicate libraries. The Cochran Mantel Haenszel (CMH) chi-square test approach may handle the data with replication, but it is a single test method, not a large-scale association test approach. This is why we need to develop a large-scale association statistical method for identifying tissue-associated or condition-associated isoform transcriptions. This method is named ranking analysis of chi-squares (RAX2). RAX2, which extends the traditional chi-square test to replicated and high-throughput transcriptomic count data of RNA reads derived from NGS, is based on comparison between a set of ranked chi-square statistics for association effects and a set of ranked null chi-square values across a set of given thresholds and estimation of a false discovery rate (FDR) profile.

## MATERIALS AND METHODS

### Cell lines and stimulation

Human primary CD4 T cells were obtained from buffy coats derived from the Gulf Coast Regional Blood Bank via the EasySep Negative Selection Kit (Stem Cell Technologies) as per manufacturer's instructions. Purity (>90%) was assessed via flow cytometry for CD3 and CD4 (UCHT1 and RPA-T4, respectively). Cells were maintained in RPMI (ATCC) with 10% fetal bovine serum supplemented with 10 mM HEPES pH 7.4, nonessential amino acids, 2 mM L-glutamate, 50 μM β-mercaptoethanol, 100 units/ml penicillin and 100 μg/ml streptomycin (all from Gibco/Life Technologies). T-cells were stimulated with plate-bound antibodies (1 μg/ml anti-CD3 (OKT3 – eBiosciences), 5 μg/ml anti-CD28 (CD28.2 – BD Pharmingen). Activation of T-lymphocytes was verified via flow cytometric detection of CD69 expression (FN-50) 16 h after stimulation, and cells were harvested at 48 h.

### High-throughput sequencing library generation

Total RNA was harvested from resting and stimulated cells with Trizol reagent (Life Technologies) as per manufacturer instructions. Polyadenylated RNA was isolated with the Poly(A)-Purist MAG (Ambion/Life Technologies) kit as per manufacturer instructions. High-throughput sequencing libraries were generated essentially as described (2), with the exception that ‘barcoded’ linkers were used to facilitate multiplexing. Libraries were sequenced via 50 bp paired-end sequencing on an Illumina GAIIx.

### Library processing and mapping

Paired end reads were mapped to the hg18 build of the human genome using the paired-end mapping module of BWA (31), default alignment stringency and requiring that each read be mapped in a proper pair. To rescue reads crossing splicing junctions, nonmapping reads were remapped to the UCSC KnownGene reference and projected back to the hg18. Individual reads were condensed into tags based on their 3′ coordinate using a sliding window of 20 nucleotides and using the frequency-weighted median 3′ coordinate as the tag identifier. Tags were then filtered by using a progressive filtering strategy to assess adenosine and guanine composition in the five, ten and fifteen bases followed the tag-mapping site. Tags were assigned to individual transcription units based on UCSC KnownGene annotations. For each transcription unit, the aggregate tags mapped to the unit were ranked based on frequency. Tags were extracted from the highest to the lowest frequency until the extracted tags represented greater than 90% of the aggregate frequency for the gene. The remaining tags were discarded. Libraries were normalized using a negative binomial model within the DEseq package (21). For ‘gene’ analysis, the summed frequency of all tags mapped to each transcription unit was considered as a single entity.

### RAX2 packages

The methods described in this paper, including estimations of null chi-square distribution and FDR, are implemented in software package RAX2. RAX2 was written in R. The current version of RAX2 is designed to analyze count data in two distinct states and the results are output in MS DOS csv format. Performance of RAX2 and the other statistical analysis process are given in Supplementary User Guidance for RAX2. The RAX2 package can be found in Supplementary Package or Bioconductor.

### Models

**Figure 2.**
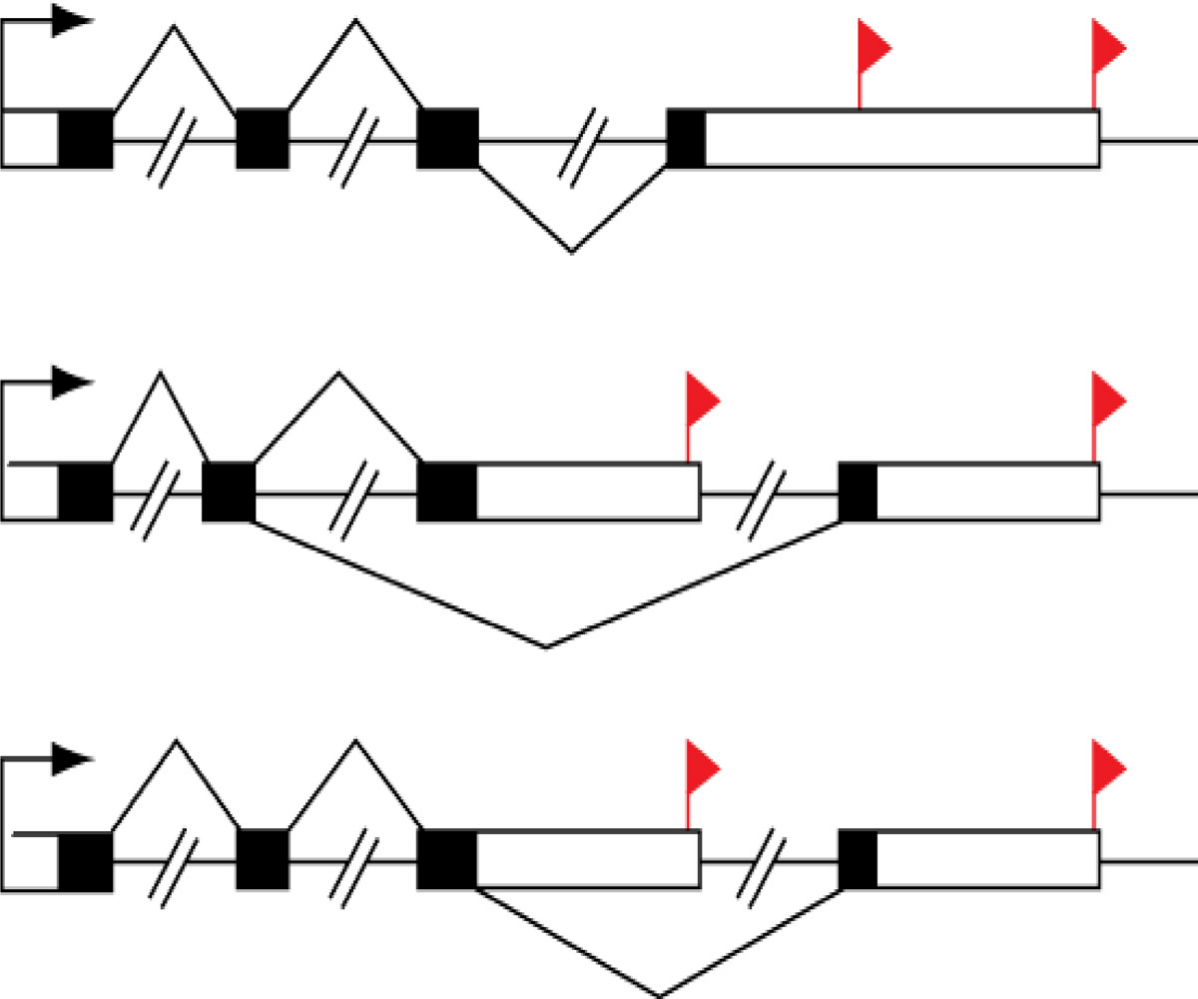
Transcription unit structures and different 3′ UTR isoforms. A majority of mammalian transcription units are characterized by alternative cleavage and polyadenylation (ACP). The majority of these contain multiple cleavage and polyadenylation sites in their terminal exon (top), impacting untranslated region identity without changing the coding sequence. Transcription units may also be characterized by mutually exclusive terminal exon structure (middle) or composite terminal exon structure (bottom). In the latter two cases, ACP is coupled to mRNA splicing, and both the coding sequence and the untranslated region are impacted. Black box: exon, white box: untranslated region (UTR), red flag: poly(A) site and -//-: intron.

**Figure 3.**
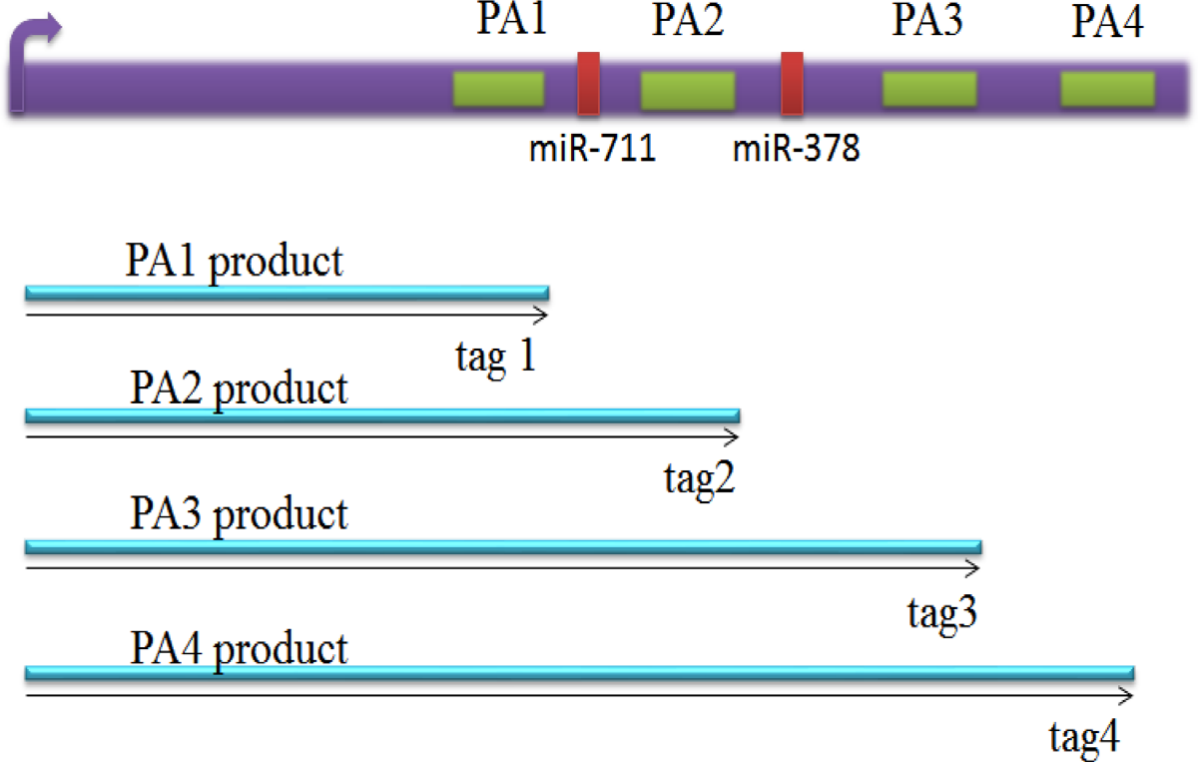
A model for multiple poly(A) sites or tags within genes. mRNA of Hsp70.3 is a typical model for multiple poly(A) sites (green boxes) and microRNA sites (red boxes). It has a common start point (promoter 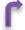), four poly(A) sites (PA1, PA2, PA3 and PA4) in 3′ UTR coding for several mRNA isoforms that are different in their 3′ end. Alternative polyadenylation changes the length of the 3′ UTR and it also can change microRNA binding sites in 3′ UTR. While microRNAs tend to repress translation and promote degradation of the mRNAs they bind to. Since transcription products are one-to-one corresponding to poly(A) sites, we define the transcription products as tags.

For convenience, our discussion about RAX2 is based on ACP sites within genes. ACP can occur concomitantly with or independently of alternative splicing (Figure 2). ACP independent of alternative splicing, that is, all cleavage and polyadenylation (poly(A)) sites are in the terminal exon of the transcription unit, results in a transcription unit with a tandem untranslated region (tandem UTR). Both alternative splicing of terminal exons and tandem UTR usage are visible for directed 3′ end sequencing methodologies. Since either process has the potential to alter 3′ UTR identity, we do not differentiate between them in this analysis. For a given transcription unit, transcript variants derived from the first poly(A) site (poly(A) site 1) are assumed to have a transcript length from the transcriptional start position to poly(A) site 1. Similarly, transcript variants with poly(A) sites 2 and 3 are also assumed to be derived from the transcriptional start position to poly(A) sites 2 and 3, respectively. Therefore, within such a transcription unit, the transcript isoforms are one-to-one corresponding to poly(A) sites. For the sake of convenience, we refer to the transcript variant at poly(A) site 1 as tag1. Similarly, the transcript variants at poly(A) sites 2, 3 and 4 are also defined as tags 2, 3 and 4, respectively (Figure 3). In general, within gene *g* (*g* = 1,.., G), *Z_g_* alternative poly(A) sites (*Z_g_* is number of poly(A) sites within gene *g*) correspondingly have *Z_g_* alternative tags. For example, gene AARS2 on chromosome 6 has four poly(A) sites(*Z_g_* = 4) and hence its mRNA transcription unit (called gene) has four isoforms or tags (see columns gene name and pos (position) in Supplementary Table S1). Supplementary Table S1 lists such count data of reads of tags within genes where columns 1–7 list information of tags or poly(A) sites within genes: tagid, geneid, name, DNA strand, position on transcription unit and annotation; columns 8–10 list count data of cells in normal and stimulated states. The data were normalized so that they look decimal. Here, we let $n_{gzjv}$ be count of tag *z* (z = 1,…, $Z_g \ge 2$) within gene *g* (*g* = 1, 2,… *G*) in cell state *j* in replicate *v* (*v* = 1,…, *r*). Our concern here is if change in count of a tag *z* at a special poly(A) site *within* a gene of interest is associated with change in condition (cell state) in the context of a discontinuous process. For the convenience, we focus on two states: treatment state (TS) and normal state (NS). We set *j* = 1 for NS and *j* = 2 for TS. To find if the transcription variation of an individual tag is associated with change in cell state, we need to compare the count of tag *z* to that of all the other tags (denoted by $^{\frown}\kern-6pt{z}$) within gene *g*. Therefore, a tag or poly(A) site has two states: z and $^{\frown}\kern-6pt{z}$. We set *i* = 1 for z and *i* = 2 for $^{\frown}\kern-6pt{z}$. Thus count $n_{gzjv}$ in the raw data table can be converted into two-by-two table count $n_{gzijv}$ where $n_{gzijv}$ is count of RNA reads of tag *z* within gene *g* in tag state *i* in condition *j* in replicate *v* (*v* = 1, 2, …, *r*) and there are $Z_g r$ two-by-two table datasets ($n_{gz11v}$, $n_{gz12v}$, $n_{gz21v}$, $n_{gz22v}$) for gene *g*. Since our interest is in regard to tag *z* (or poly(A) site *z*) within gene *g* instead of gene *g* itself, totally we have $S = \sum\nolimits_{g = 1}^G {Z_g }$ tags or poly(A) sites in all genes of study. To implement ranking analysis of all tags, we combine subscripts *g* and *z* into *s* where *s* = 1, …, S. Thus, each tag *s* has two states (*s* and $^{\frown}\kern-6pt{s}$). We still set *i* = 1 for tag state *s* and *i* = 2 for tag state $^{\frown}\kern-6pt{s}$. The two-by-two table count table dataset ($n_{gz11v}$,$n_{gz12v}$,$n_{gz21v}$,$n_{gz22v}$) is then rewritten as ($n_{s11v}$,$n_{s12v}$,$n_{s21v}$,$n_{s22v}$).

Since there are replicate observations for each tag, each count consists of two components: the association effect and random noise. Thus, a model for each count in such a two-by-two table is

(1) \begin{equation*} n_{sijv} = n_{sij} + e_{sijv} \end{equation*}

where $n_{sij}$ is count of RNA reads of tag *s* due to association effect between state *i* of poly(A) site *s* and cell state *j* and $e_{sijv}$ is count of RNA reads due to a special noise between state *i* of poly(A) site *s* and cell state *j* in replicate observation *v*. Since $n_{sijv}$ follows Poisson distribution (21,24) or negative binomial distribution (21,24,25) or binomial distribution (32), $e_{sijv}$ may also follow one of these distributions. But we here do not concern distribution of $e_{sijv}$ because we do not need to separate it from $n_{sijv}$. Let $f_{sij}$ and $f_{sijv}$ be frequencies of counts *n_sij_* and *e_sijv_*, respectively. As $n_{sij}$ is count of RNA reads of tag *s* in state *i* and in cell state *j*, $n_{sij} = n_s f_{sij}$ and $e_{sijv} = n_s f_{sijv}$ where $n_s$ is total count of RNA reads of tag *s within g* across two cell states and two tag states over all replicate libraries. Thus, model (1) may be rewritten as

(2) \begin{equation*} n_{sijv} = n_s f_{sij} + n_s f_{sijv} . \end{equation*}

### Chi-square statistics

For poly(A) site *s*, the mean count ($\bar n_{sij}$) of RNA reads over *r* replicates consists of association effect $n_{sij}$ between site state *i* and cell state *j* and mean noise($\bar e_{sij}$):

(3) \begin{equation*} \begin{array}{*{20}l} {\bar n_{sij} = \frac{1}{r}\sum\limits_{v = 1}^r {n_{sijv} } = }  \\ {n_s f_{sij} + \frac{{n_s }}{r}\sum\limits_{v = 1}^r {f_{sijv} } = n_s f_{sij} + n_s \bar f_{sij} = n_{sij} + \bar e_{sij} }  \\ \end{array} \end{equation*}

where $f_{sij}$ is the frequency of association effect $n_{sij}$ between state *i* of tag *s* and cell state *j*, $f_{sijv}$, frequency of a special noise in replicate observation *v* and $\frac{1}{r}\sum\nolimits_{v = 1}^r {f_{sijv} } = \bar f_{sij}$. For poly(A) site *s*, $\bar n_{sij}$ is expected as

(4) \begin{equation*} E(\bar n_{sij} ) = E(n_{sij} ) + E(\bar e_{sij} ). \end{equation*}

(See Supplementary Note S1 for detail), that is, expectation of mean count ($\bar n_{sij}$) of RNA reads of tag *s* in tag state *i* and cell state *j* equals expectation of association effect ($n_{sij}$) plus expectation of mean noise ($\bar e_{sij}$). Thus, Pearson chi-square statistics for mean count $\bar n_{sij}$ that is estimate of association effect $n_{sij}$ is

(5) \begin{equation*} \chi _s^2 (\bar n) = \chi _s^2 (n + \bar e) \end{equation*}

(See Supplementary Note S2 for derivation). From Equation (5), we can see that $\chi _s^2 (\bar n) \ne \chi _s^2 (n)$ unless the mean of noises is zero ($\bar e = 0$), meaning that the Pearson chi-square for $\bar n_{sij}$ is not an unbiased estimate of the chi-square statistic for $n_{sij}$. For this reason, we cannot use $\chi _s^2 (\bar n)$ as standard chi-square statistic, that is, *P*-value for $\chi _s^2 (\bar n)$ obtained from a chi-square distribution has a big bias. In addition, $\chi _s^2 (\bar n) = \chi _s^2 (\bar e)$ if, for a tag *s*, there is no association effect $n_{sij}$ between tag states and cell states, say, $\bar n_{sij} = \bar e_{sij}$.

### Null chi-squares

Similarly to using within-group variance to estimate null variance, we also employ within-group chi-square to estimate null chi-square. To this end, we need to construct another contingent table in which we have site states *s* and $^{\frown}\kern-6pt{s}$ within gene *g*, and *r* replicates in cell state *j*. Set *i* = 1 for s and *i* = 2 for $^{\frown}\kern-6pt{s}$. A set of two-by-two tables (see Supplementary Note S3) is constructed with pairs of replicates and states of tag *s* in cell state *j*. Thus the null chi-square is easily estimated by $\chi _s^2 (e)$ (see Supplementary Note S3 for derivation).

### Ranking analysis of chi-squares

Since $\chi _s^2 (\bar n)$ does not have one-to-one correspondence to $\chi _s^2 (e)$, to compare them, we need to separately rank tags by amounts of $\chi _s^2 (\bar n)$ and $\chi _s^2 (e)$ from the smallest to the largest. Let $\chi _{s^*}^2 (\bar n)$ and $\chi _{s^*}^2 (e)$ be two Pearson chi-square values at position s* in the ranking linear space(*), the chi-square values are smallest at s* = 1 and largest at s* = S*. Then we can compare $\chi _{s^*}^2 (\bar n)$ to $\chi _{s^*}^2 (e)$ at position s* in a one-to-one fashion. Given threshold Δ, expression change of tag s* is declared to be associated with change in cell state if and only if

(6) \begin{equation*} \chi _{s^*}^2 (\bar n) - \chi _{s^*}^2 (e) >\Delta . \end{equation*}

### FDR estimation

In large-scale statistical analysis, we do not need to be concerned with so-called type 1 error but we must consider how to control false discovery rate (FDR) because in, for example, 10 000 hypotheses to be tested, at least 500 hypotheses would commit type I error if α = 0.05 is chosen as significance level (33). Obviously 500 hypotheses rejected by chance are not acceptable. This is why we here want to control FDR rather than type I error. A reasonable control of FDR involves a reliable estimation of FDR. Given threshold Δ, the number (*N*_Δ_) of tags whose expression changes are declared to be uniquely associated with change in cell state may consist of the number of truly positive tags, *T*_Δ_, and number of falsely positive tags, *F*_Δ_:

(7) \begin{equation*} N_\Delta = T_\Delta + F_\Delta . \end{equation*}

Therefore, FDR is expected as $FDR_\Delta = E(F_\Delta /N_\Delta )$ at threshold Δ. As *F*_Δ_ is unknown in experimental datasets, FDR must be estimated.

**Figure 4.**
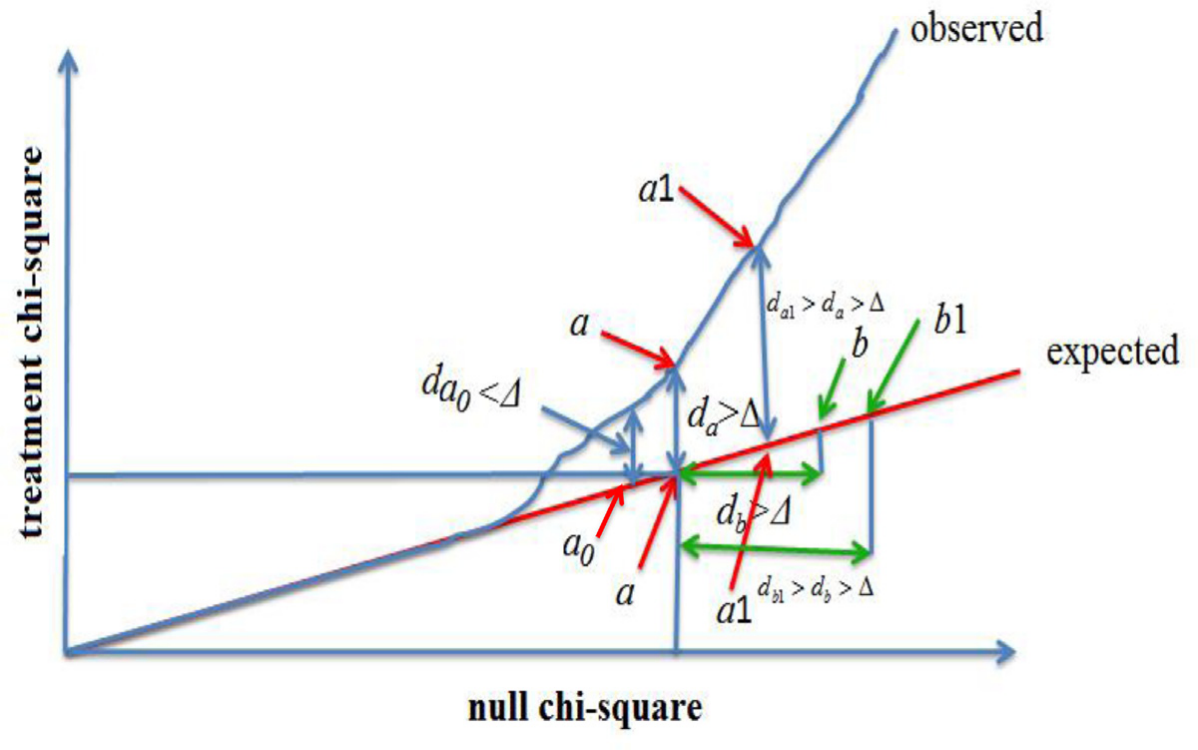
A demonstration plot of ranked treatment chi-square versus null chi-square. The expected linear plot (red line) is given by null hypotheses that the treatment chi-square is equal to null chi-square at each chi-square point. The observed linear plot is given by ranked observed treatment chi-squares versus ranked estimated null chi-squares. Given a threshold Δ, all treatment chi-squares with $d_s \ge d_a$ are declared to be significant or interested where $d_a = \chi _a^2 (\bar n) - \chi _a^2 (e) \ge \Delta$ and *s* = *a* +1,…, *G*. All null chi-squares with $d_t \ge d_b$ are defined as potential false positives with probability given in Equation (9) where $d_b = \chi _b^2 (e) - \chi _a^2 (e) \ge \Delta$ and *t* = *b*+1,…, *G*.

The existing methods for estimation of FDR are not suitable to our current chi-square statistics because, as seen above, chi-square statistics are remarkably biased against standard chi-square statistics such that *P*-values obtained from chi-square distribution are also biased. For this reason, we here propose a novel method. The principle of the method is shown in Figure 4. As seen in Figure 4, ordered and observed chi-square $\chi _{s^*}^2 (\bar n)$ more and more deviates from ordered and expected chi-square $\chi _{s^*}^2 (e)$ as chi-square value increases.

Given threshold Δ, we find that $d_a = \chi _a^2 (\bar n) - \chi _a^2 (e) >\Delta$ at position *a* in the ranking linear space (see Figure 4) is the smallest distance among all s*. We define *a* as $a_\Delta$ and $d_a$ as $d_{a_\Delta }$. All $d_{s^*} = \chi _{s^*}^2 (\bar n) - \chi _{s^*}^2 (e) >\Delta$ would be larger than or equal to $d_{a_\Delta }$ where $s^* = a_\Delta ,a_\Delta + 1$, .., *S*, that is, all tags at $s^* \ge a_\Delta$ would be declared as positive tags. So, the number of positives declared by Equation (6) is obtained by

(8) \begin{equation*} N_\Delta = S - a_\Delta + 1. \end{equation*}

In order to avoid confusion, s* in the ordered null chi-square space is replaced with t*. Likewise, for a given threshold Δ, at $t^* = b_\Delta$, we also find that $d_{b_\Delta } = \chi _{b_\Delta }^2 (e) - \chi _{a_\Delta }^2 (e) >\Delta$ is smallest among all t* (see Figure 4). All $\chi _{t^*}^2 (e) - \chi _{a_\Delta }^2 (e) >\Delta$ would be larger than or equal to $d_{b_\Delta }$. In addition, within the ranking space of $\chi _{t^*}^2 (e)$, null tag at $t^* = b_\Delta + j$ has smaller chance to be chosen as a false positive than at $t^* = b_\Delta + k$ where $j < k = 1,...,S - b_\Delta$ and the ranking space of $\chi _{t^*}^2 (e)$ varies with sample size. In other words, the probability that a null tag at position t* is chosen as false positive is determined by replicate number *r*, chi-square space, tag space S*, null chi-square values and t*. The logic relationship is that position t*in the ranking linear space is positively related to the probability that the null tag at position t*is chosen as a false-positive tag and null chi-square value is also positively related to the probability that the null chi-square is chosen as false-positive chi-square. As position t* is fixed in a given tag space, the ratio (t*/S*) would not alone fit to changeable false-positive probability from practical data; while $\chi _{t^*}^2 (e)$ is variable and depends on a practical dataset and its values at some nearby positions may not vary or very approximate, so ratio ($\chi _{t^*}^2 (e)/\chi _{S^*}^2 (e)$) is also not alone appropriately used as the false-positive probability. To obtain an accurate estimate of false-positive probability we need to combine them. A good and simple combination way is geometric mean. We use geometric mean $\sqrt {(\chi _{t^*}^2 (e)/\chi _{S^*}^2 (e))(t^*/S^*)} = \left[ {\left( {\chi _{t^*}^2 (e)t^*} \right)/\left( {\chi _{S^*}^2 (e)S^*} \right)} \right]^{0.5}$ to estimate the probability that a null tag at position t* is chosen as false positive. Additionally, in a single hypothesis test the *P*-value of statistic is negatively related to sample size, similarly, the probability that the null chi-square at position t* is chosen as false-positive chi-square is also negatively related to sample size because increment of sample size would let space of $\chi _{t^*}^2 (e)$ be shrunken. On the other hand, $\chi _{S^*}^2 (e) = \max (\chi _s^2 (e))$ is positively related to the probability. We therefore replace 0.5 with $x = \left( {2r/\chi _{S^*}^2 (e)} \right)^2$. Thus, probability that a tag at position t* is chosen as a false-positive tag is given by

(9) \begin{equation*} p(t^* |\chi _{t^*}^2 (e)) = \left( {\frac{{\chi _{t^*}^2 (e)t^* }}{{\chi _{S^*}^2 (e)S^*}}} \right)^x . \end{equation*}

The number of false positives in $N_\Delta$ findings is estimated by

(10) \begin{equation*} F_\Delta = \sum\limits_{t^* = b_\Delta }^{S^*} {p(t^* |\chi _{t^*}^2 (e))} . \end{equation*}

When $x \to 0$, $p(t^* |\chi _{t^*}^2 (e)) \to 1$ such that $F_\Delta \to S - b_\Delta + 1$. This is an extreme case in which FDR is very conservatively estimated (see Supplementary Figure S1A1, A2 and A3). In general, larger sample sizes would have a smaller $\chi _{S^*}^2 (e)$, leading to a larger *x* value that causes a smaller $p(t^* |\chi _{t^*}^2 (e))$. This is agreeable with the fact that larger sample sizes would have a smaller FDR. With $F_\Delta$, FDR for $N_\Delta$ findings declared by chi-square tests at threshold Δ is estimated as

(11) \begin{equation*} FDR_\Delta = \frac{{F_\Delta }}{{N_\Delta }}. \end{equation*}

## RESULTS

### Simulation evaluation of RAX2

We use the following steps to generate the null data of *S* tags with *r* replicates based on the real data in a group:

Step1: Calculate four variances and four means from two-by-two tables over *r* replicates for each tag within a gene.

Step2: Choose randomly one variance and one mean with equal probability for each tag within a gene.

Step 3: Generate a two-by-two random null or baseline count data with *r* replicates using negative binomial pseudorandom generator in R environment with the mean as mu and the variance as dispersion (size).

Step 4: Repeat steps 1–3 until *g* = *G*.

We then adopted uniform and normal pseudorandom generators to generate count data with association effects:

(12) \begin{equation*} n_{gzij} = U_{gzij} \frac{{N_{gzi} N_{gzj} }}{{N_{gz} }} \end{equation*}

where $N_{gzi}$ and $N_{gzj}$ are normal variables with *σ* = 50 and mean = 100 (these values are arbitrary because associate effect does not depend on individual values) within gene g, $N_{gz} = \sum\nolimits_{i = 1}^2 {N_{gzi} } + \sum\nolimits_{j = 1}^2 {N_{gzj} }$, and $U_{gzij} = U_{gzi} U_{gzj}$ where $U_{gzi}$ and $U_{gzj}$ are uniform variables, $0 < U_{gzi} \le 1$ and $0 < U_{gzj} \le 1$, *i* = 1 and 2, *j* = 1 and 2, *z* = 1, …, *Z*_g_. The association effect $n_{gzij}$ is randomly assigned to 10, 20 and 30% of null tags (the null data above). Using these simulated datasets, we compared the estimated FDR given by a statistical method to its true FDR across a set of given thresholds ($\Delta = U\chi _{S^*}^2 (e)$, $0 \le U \le 1$).

Supplementary Figure S1 summarizes these results in the simulated scenarios where 10% (A1 and B1), 20%, (A2 and B2) and 30% (A3 and B3) of tags have association effects on transcription with cell states and *r* = 6, *x* = 0 and $x = \left( {2r/\chi _{S^*}^2 (e)} \right)^2$ where $\chi _{S^*}^2 (e) = \max (\chi _s^2 (e))$. With *x* = 0 (Supplementary Figure S1A1–3), FDR was significantly overestimated in all three given scenarios such that many tags with true association effects would be missed at FDR <0.05 level. In contrast, with $x = \left( {2r/\chi _{S^*}^2 (e)} \right)^2$ (Supplementary Figure S1B1–3), the estimated FDR curve is slightly higher than the true one in each of these three scenarios, finding tags with a true association effect at a given threshold with higher power. Supplementary Figure S1C1–3 is obtained with $x = \left( {2r/\chi _{S^*}^2 (e)} \right)^2$ from the simulated data with *r* = 3 and 10, 20 and 30% of tags having association effect, respectively. The fact that these FDR curves are very similar to those in Supplementary Figure S1B1, B2 and B3 indicates that our estimate of FDR profile with $x = \left( {2r/\chi _{S^*}^2 (e)} \right)^2$ is absolutely conservative but close to its true one across a set of given thresholds in these scenarios.

### Comparison with Pearson chi-square, Fisher exact test and CMH chi-square test approaches

In order to show strong advantages of our method, we applied Fisher exact test, Pearson chi-square test and CMH chi-square test approaches to the simulated data and used Benjamini-Hochberg (BH) multiple-testing procedure (34,35) and q-value (36,37) to estimate FDR. Since the Fisher exact test and Pearson chi-square methods do not work on the replicated data, we utilized mean counts over *r* replicate libraries to construct a two-by-two table for each tag. These association test methods were performed in R packages (fisher.test, chsq.test and CMH.test), the BH procedure was conducted in R package p.adjust with method = BH. Package qvalue (36,37) was downloaded from Bioconductor and its qvalue.gui() was used to calculate q-values. The *P*-values were obtained from application of these three approaches to our simulated data of 15391 tags with three replicates among which 10% of tags were given with association effects. The three sets of 15 391 *P*-values for Fisher exact tests, Pearson chi-square tests and CMH chi-square tests were listed in Supplementary Table S2 and adjusted by HB-procedure and q-value method. Supplementary Figure S2 displays the results of plotting estimated against true FDR values across all cutoff points. One can find from Supplementary Figure S2 that FDRs are completely overestimated by BH-procedure and q-value approach. The same case was also found in the simulated data with five replicates (data not shown). As indicated above, Pearson chi-square based on mean counts over replicates is not unbiased and the P-value for such chi-square obtained from a chi-square distribution is greatly biased against its true value (See Supplementary Table S2). So BH and q-value procedures are not available to adjust such biased *P*-value profiles.

### RAX2 analysis of the primary T-cell data

**Figure 5.**
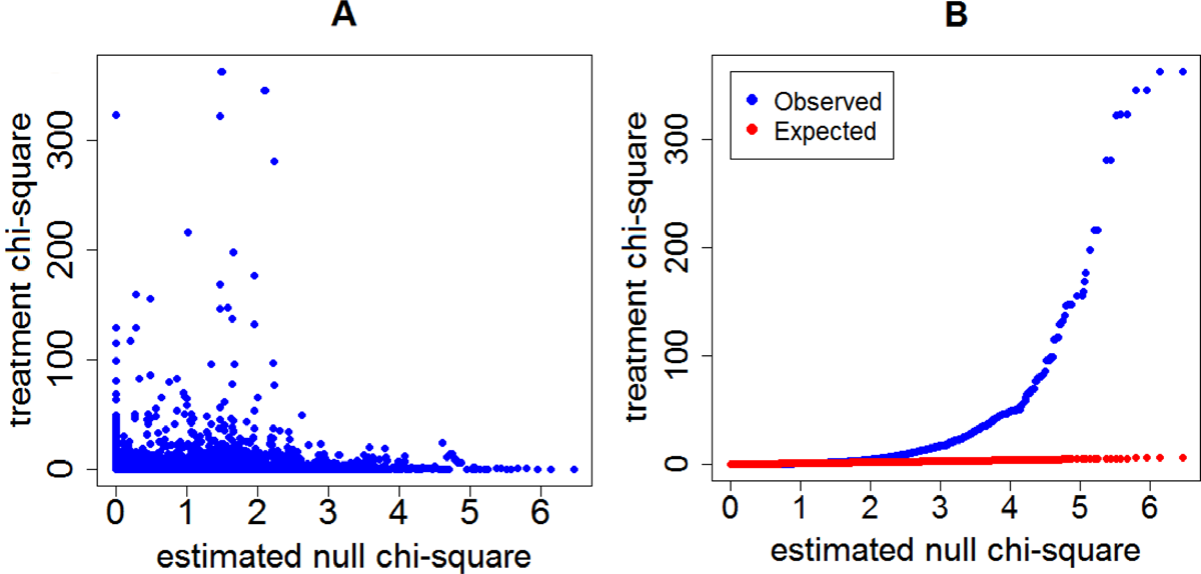
Scatter and linear plots of treatment chi-squares versus estimated null chi-squares. (**A**) Scatter plot of treatment chi-squares against estimated null chi-squares derived from primary experimental data. Over 80% of treatment chi-squares fall in the null chi-square distribution. (**B**) Linear plot of ranked treatment chi-squares against ranked estimated null chi-squares. The observed linear dots (blue line) were significantly deviated from the expected linear dots (red line) across the estimated null chi-square distribution.

We then assessed the performance of RAX2 on 3p-seq datasets derived from resting and stimulated primary human CD4^+^ T cells (Supplementary Table S1). After normalizing and filtering, the data contain 16247 tags scattered in 10160 transcription units or genes. Transcription units with a single tag were filtered and the remaindered 9899 tags scattered in 3812 genes were available for RAX2 analysis. We performed classical chi-square and the BH- procedure on our primary T-cell transcriptomic data. As expected in Supplementary Figures S2, only 747 tags were found to be associated with cell states at FDR < 0.05 (see Supplementary Table S4). We therefore applied RAX2 to the real data. The estimated null chi-square value falls in a range of 0 to 6.7 (Figure 5A). The treatment chi-square falls in interval between 0 and 363, which is larger than that in the estimated null chi-square distribution. The observed linear dots are deviated from the expected linear dots when the null chi-square is larger than 2.5 (Figure 5B).

**Figure 6.**
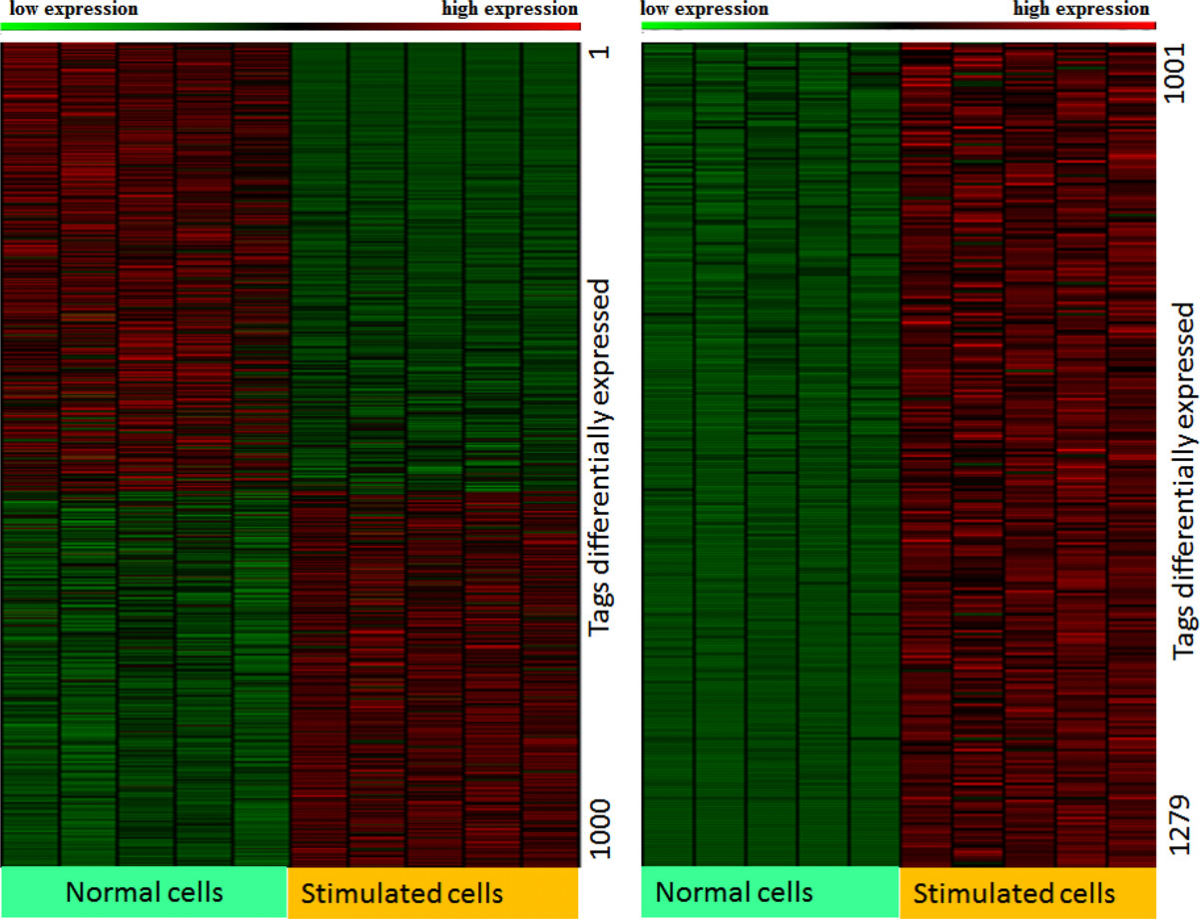
Heatmap of tags differentially expressed between two conditions detected by RAX2. The heatmap was made with the data of 1279 tags with fold change (average of tag expressions in stimulated cells/average of tag expressions in normal cells or average of tag expressions in normal cells/average of tag expression in stimulated cells)> 1.4 selected from 1610 associated tags detected by RAX2.

**Figure 7.**
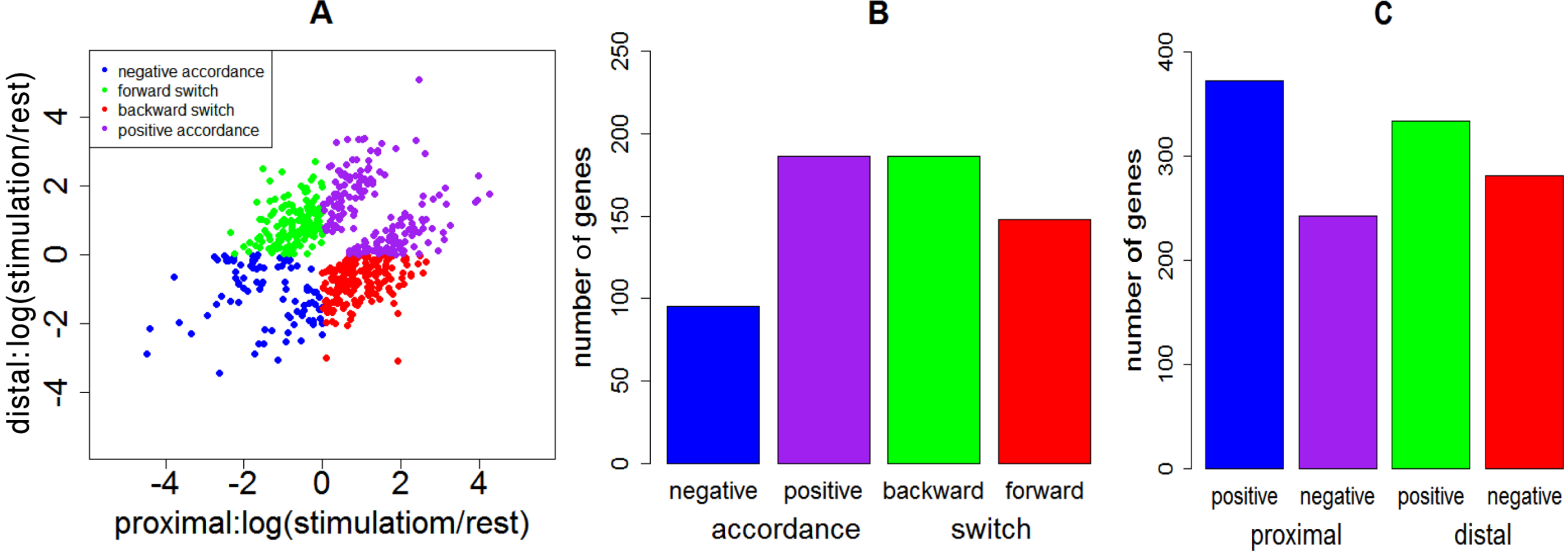
Two-way scatter plot for four association patterns of transcription of two tags within genes between two cell states. A two-way scatter plot displays distributions of scatter dots in four phases: Phase II for forward switch, phase IV for backward switch, phase I for positive accordance and phase III for negative accordance. (**A**) Plot of proximal tags versus distal tags where coordinates *x* and *y* are differences between ratios of counts of tags in stimulation and those in rest state. The ratio = sum of transcription counts of a tag over all replicates in a cell state / sum of transcription counts of this tag over all replicates and all cell states. (**B**) Numbers of genes with four transcription patterns of proximal and distal tags. (**C**) Numbers of genes with proximal tags positively and negatively responses to stimulation and numbers of genes with distal tags positively and negatively response to stimulation

**Figure 8.**
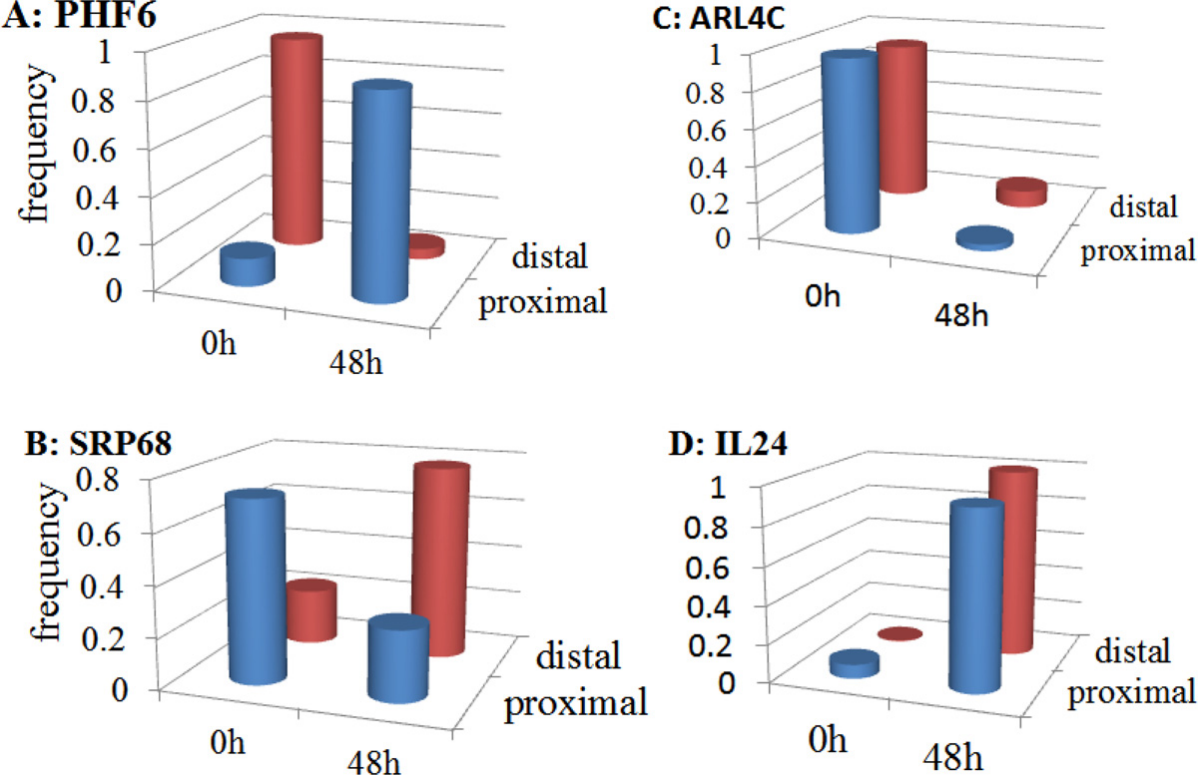
Examples for association patterns of transcriptional representation of two tags within genes between two cell states. Gene products shown here contain two tags defined by usage of distal and proximal poly(A) sites. Association between relative expression of two tags within genes and the cell states, detected by RAX2, shows backward switch (**A**), forward switch (**B**), negative accordant changes (**C**) and positive accordant changes (**D**). y axis corresponds to ratio of count sum of a tag over all replicate libraries in a cell state (0 h or 48 h post stimulation) to the sum of the tag over all replicate libraries across all cell states.

The results obtained by applying RAX2 to our real transcriptomic data are illustrated in Supplementary Tables S3–S5. At FDR < 0.05, 1610 (16.3%) tags were found to be associated in transcription with antigen receptor stimulation (Supplementary Table S3). Among 1610 tags, 1279 have fold change >1.4 or < 0.715. Heatmap shows that these 1279 associated tags also displayed strong differential expression between rest and stimulation (Figure 6). We chose genes with two tags identified at FDR < 0.05 (see Supplementary Table S6), and plotted logarithms of the ratio of transcription relative frequencies of proximal tags in stimulation to those in normal condition against those of distal tags and observed four patterns of transcription variation: forward and backward ‘switch’ changes; positively and negatively accordant changes (Figure 7A) where forward switch is defined when high expression is switched from proximal site to distal site and backward switch when high expression is switched from distal site back to proximal site. We call such an association expression positively accordant expression when expression of two tags is highly raised by stimulation or negatively accordant expression when both come down due to stimulation. In our data, genes with forward switch were many fewer than genes with backward switch (148 versus 186), implicating that under stimulation, usages of many distal poly(A) sites switched to proximal poly(A) sites. In addition, positively accordant genes were many more than negatively accordant genes (186 versus 85) (Figure 7B). This means that stimulation significantly promoted transcription of tags. Figure 7C shows that proximal tags positively associated with stimulation are significantly more than distal tags positively associated with stimulation (372 versus 334). This result indicates that proximal poly(A) sites were preferably used under stimulation. This is consistent with the previous studies (18–20). Sandberg *et al*. (17) found that tandem UTR length is highly negatively correlated to proliferation ratio. That is, proliferative cells more use proximal poly(A) sites. Mayr and Bartel (16) also observed that cancer cells preferably used short 3′ UTR while tissue cells more used long 3'UTR because cancer cells have high proliferation ratio but tissue cells are highly differentiated. CD3/CD28 costimulation triggered a series of physiological activations and expansion (growth) of T-cells. Figure 8 displays examples for forward and backward switches, positively and negatively accordant changes. Figure 8A is backward switch between two tags within gene PHF6 (PHD finger protein 6) potentially playing a role in transcription regulation. Stimulation made high transcription level switched from distal poly(A) site backward to proximal poly(A) site. In gene SRP68 (Signal Recognition Particle of 68 kDa, a ribonucleoprotein complex), the transcription pattern of two tags (Figure 8B) is completely inverse with that in gene PHF6, called forward switch. Figures 8C and 8D show negatively and positively accordant transcription of proximal and distal tags in genes ARL4C (ADP-Ribosylation Factor-Like 4C) and IL24 (interleukin 24). Stimulation triggered high transcription of two tags in gene IL24 but strongly suppressed transcription of proximal and distal tags in gene ARL4C.

In our result, 162 genes were found to have three poly(A) sites whose usages were associated with stimulation (Supplementary Table S8). Supplementary Figure S3 displays three scatter plots of these genes with proximal tags versus middle tags (Supplementary Figure S3A1), proximal tags versus distal tags (Supplementary Figure S3A2) and middle tags versus distal tags (Supplementary Figure S3A3). In plot of proximal versus middle tags (Supplementary Figure S3A1), accordantly changed tags were many more than switched tags (47 versus 9 in Supplementary Figure S3B1). Among accordantly changed tags, proximal and middle tags positively responding to stimulation are more than those negatively responding to stimulation (Supplementary Figure S3C1). This result suggests that stimulation indeed raised transcription of tags but did not significantly trigger transcription switch between proximal and middle poly(A) sites. However, Supplementary Figures S3A2 and S3A3 definitely show that stimulation remarkably increased transcription switch from distal poly(A) sites backward to middle poly(A) site (Supplementary Figure S3B2) or backward to proximal poly(A) sites (Supplementary Figure S3B3) while positively and negatively accordant transcriptions between proximal and distal poly(A) sites and between middle and distal poly(A) sites became very weak. In genes with proximal and middle poly(A) sites, positive tags were more than negative tags but in genes with distal poly(A) sites, negative tags were more than positive tags (Supplementary Figure S3C2–3). These results strongly indicate that, as seen in genes with two tags, in genes with three tags, CD3/28 cosimulation resulted in more usage of shorter tags.

## DISCUSSION

Transcriptional profiling via NGS technologies has underscored the complexity of transcript isoform variation in mammalian cells. The extent of this variation, whether the identity and function of a protein product (e.g. alternative splicing) or the visibility of the gene product is altered due to the posttranscriptional regulatory machinery (e.g. ACP), has been broadly appreciated. It is of great interest to leverage newer technologies to globally assess the impact of these processes on human disease, particularly since several individual examples in which dysregulation of either process contributes to human disease exist (5).

The identification of relative expression changes of individual mRNA isoforms derived from a single transcription unit can in theory be performed by considering each of these isoforms from the transcription unit as a single entity and applying statistical approaches DESeq (21), baySeq (22), edgeR exact test (23,24), edgeR GLM (25), DEXSeq (26), Cuffdiff (27,28), DiffSplice (29) and SplicingCompass (30) to determine whether a given isoform is differentially expressed between two different cell states. However, these approaches do not directly assess whether isoforms within genes are associated with the conditions in transcription. Valid statistical approaches for doing so are Pearson chi-square test and Fisher exact test, but these methodologies cannot currently use information of variation in replicate libraries. As seen in Supplementary Appendix B, the Pearson chi-square of mean count over all given replicates is biased because mean noise cannot be excluded. CMH chi-square test approach can be applied to repeat two-by-two table data or stratified count data and indeed its power is significantly higher than Pearson chi-square tests and Fisher Exact tests (see Supplementary Table S2), so it will be promised to extend our RAX2 to CMH and to develop a multiple CHM test approach.

Therefore, we need to develop a novel chi-square test methodology. Within the context of NGS datasets where individual transcription units have a potential to be characterized by the production of multiple mRNA isoforms, it is not clear what the relationship is between replication and the *P*-values of chi-square statistics. To avoid this puzzle issue, a nonparametric approach is preferably chosen. This approach requires that an observed Pearson chi-square profile be compared to the null Pearson chi-square profile given a threshold. Since null chi-square profile does not exist, it must be estimated. In our methodology, we adopt a manner similar to estimation of null variance from replicate within-cell data to estimate the null chi-square profile. To choose appropriate threshold, we need to estimate FDR cutoff within which the findings declared by comparison between observed and null chi-square profiles are believed to be reliable.

While there are several alternative approaches for estimation of FDR, for example, the Benjamini-Hochberg procedure (34,35), the Benjamini-Liu procedure (38) and the Pounds–Cheng procedure (39), Tan-Xu multiprocedures (40), these FDR estimators are based on *P*-value profile and are not suitable to our nonparametric method. The permutation-based estimator developed by Tusher *et al*. (33) has been shown both in theory and in simulation to be handicapped by bias in small sample sizes (41,42). Storey and Tibshriani (37) proposed a q-value as a new FDR estimator. Similarly, the q-value approach is also based on *P*-values (36,37). The other ranking analysis methods (41–43) are designed to be applicable to continues data sets or cannot otherwise be used to this context. To accurately estimate FDR for the chi-square findings, we here proposed a novel approach. Our approach is based on a simple principle: given a threshold, tags were declared to be associated with conditions by comparing treatment ranking chi-square profile to null ranking chi-square profile and number of false tags associated with conditions was determined by comparing null chi-square at low ranking position to that at higher ranking position. For a given threshold Δ, a tag at $t^* = b_\Delta + k$ in a linear space of the null chi-squares has growth of probability to be declared as a false positive with increment of *k*. As seen in Figure 4, if a treated chi-square distribution falls into the null chi-square distribution, then we cannot find $a_\Delta$ value except for $a_\Delta$ = 1 across a set of given thresholds, and hence we also cannot find $b_\Delta$ value except for $b_\Delta = S$. Thus, $N_\Delta = 0$,$F_\Delta = 0$, and FDR cannot be defined in such a case. Supplementary Figure S1 shows that estimated FDR is larger than its true FDR when threshold is small but it tends to be very close to its true value as threshold increases. This property guarantees that estimation of FDR is conservative and reliable at any threshold level.

## SUPPLEMENTARY DATA

Supplementary Data are available at NAR Online.

SUPPLEMENTARY DATA
