# Supplementary material for: RAX2: a genome-wide detection method of condition-associated transcription variation
Source: Nucleic Acids Res. 2015 May 7;43(15):e96. doi: 10.1093/nar/gkv411 (PMC4551904; doi:10.1093/nar/gkv411)
Supplement: SUPPLEMENTARY DATA [file supp_43_15_e96__index.html]

RAX2: a genome-wide detection method of condition-associated transcription variation — SUPPLEMENTARY DATA 

# RAX2: a genome-wide detection method of condition-associated transcription variation

## SUPPLEMENTARY DATA

- SUPPLEMENTARY DATA
- SUPPLEMENTARY DATA
- SUPPLEMENTARY DATA
- SUPPLEMENTARY DATA
- SUPPLEMENTARY DATA
- SUPPLEMENTARY DATA
- SUPPLEMENTARY DATA
- SUPPLEMENTARY DATA
- SUPPLEMENTARY DATA
- SUPPLEMENTARY DATA
