# Supplementary material for: RAX2: a genome-wide detection method of condition-associated transcription variation
Source: Nucleic Acids Res. 2015 May 7;43(15):e96. doi: 10.1093/nar/gkv411 (PMC4551904; doi:10.1093/nar/gkv411)
Supplement: SUPPLEMENTARY DATA [file supp_gkv411_nar-00147-met-n-2015-File004.docx]

**Note S1**

Let $\bar{n}_{si}$and $\bar{n}_{sj}$ be means of counts of RNA reads at site s in tag state *i* and cell state *j* over *r* replicate libraries, respectively, $\bar{n}_{sij}$be mean count of RNA reads at size s in tag state *i* and cell state *j* over replicate libraries and $\bar{n}_{s}$be mean count of RNA reads at site s over *r* replicate libraries. Expectation of $\bar{n}_{sij}$is

where , , and

where $p_{si}=\sum_{j=1}^{2} f_{sji}$, $q_{sj}=\sum_{i=1}^{2} f_{sji}$ , $\bar{\varphi}_{sj}=\sum_{i=1}^{2} \frac{1}{r}\sum_{v=1}^{r} f_{sijv}$,$\bar{\phi}_{sj}=\sum_{i=1}^{2} \frac{1}{r}\sum_{v=1}^{r} f_{sijv}$.

**Note S2**

where $E(\bar{e}_{sij})$=0.

**Note S3**

$$\begin{matrix} \begin{matrix} & & replicates & \\ sitestate & 1 & \cdots& r \\ 1 & n_{1j1} & \cdots& n_{1jr} \\ 2 & n_{2j1} & \cdots& n_{2jr} \end{matrix}\Rightarrow\begin{matrix} \\ \mathop{\begin{matrix} & 1 & 2 & sum \\ 1 & n_{1j1} & n_{1j2} & T_{1j12} \\ 2 & n_{2j1} & n_{2j2} & T_{2j12} \\ sum & T_{j1} & T_{j2} & T_{j12} \end{matrix},\begin{matrix} \\ \end{matrix}\cdots,\begin{matrix} \\ \end{matrix}\begin{matrix} & 1 & r & sum \\ 1 & n_{1j1} & n_{1jr} & T_{1j1r} \\ 2 & n_{2j1} & n_{2jr} & T_{2j1r} \\ sum & T_{j1} & T_{jr} & T_{j1r} \end{matrix}}\limits\end{matrix}, \\ \\ \begin{matrix} \\ \\ \end{matrix}\begin{matrix} & 2 & 3 & sum \\ 1 & n_{1j2} & n_{1j3} & T_{1j23} \\ 2 & n_{2j2} & n_{2j3} & T_{2j23} \\ sum & T_{j2} & T_{j3} & T_{j23} \end{matrix},\begin{matrix} \\ \end{matrix}\cdots,\begin{matrix} \\ \end{matrix}\begin{matrix} & 3 & 4 & sum \\ 1 & n_{1j3} & n_{1j4} & T_{1j34} \\ 2 & n_{2j3} & n_{2j4} & T_{2j34} \\ sum & T_{j3} & T_{j4} & T_{j34} \end{matrix},\begin{matrix} \\ \end{matrix}\cdots,\begin{matrix} \\ \end{matrix}\begin{matrix} & r-1 & r & sum \\ 1 & n_{1j(r-1)} & n_{1jr} & T_{1j(r-1)r} \\ 2 & n_{2j(r-1)} & n_{2jr} & T_{2j(r-1)r} \\ sum & T_{j(r-1)} & T_{jr} & T_{j(r-1)r} \end{matrix} \end{matrix}$$

$$\chi_{s}^{2}(e)=\frac{1}{2r(r-1)}\sum_{j=1}^{2} \sum_{i=1}^{2} \sum_{h=k+1}^{r} \sum_{k=1}^{r-1} \left[\frac{\left(n_{sijk}-E(n_{sijk})\right)^{2}}{E(n_{sijk})}\right.+\left.\frac{\left(n_{sijh}-E(n_{sijh})\right)^{2}}{E(n_{sijh})}\right]$$

where $E(n_{sijk})=\frac{T_{sjk}T_{sijkh}}{T_{sjkh}}$and $E(n_{sijh})=\frac{T_{sjh}T_{sijkh}}{T_{sjkh}}$. $T_{sjh}$and $T_{sjk}$are sums of counts of RNA reads at site *s* in replicate combinations *h* and *k* in cell state *j* over tag state *i*, respectively; *T_sijkh_* is sum of counts of RNA reads at site *s* in state *i* (row *i*) in cell state *j* over replicates *h* and *k*, and *T_sjkh_* is total counts of reads at site s in cells state *j* over state *i* and replicates *h* and *k*. In the above equation, square parentheses have two terms, number of replicate pairs is $r(r-1)$, so total number of replicate pairs is $2r(r-1)$.

**Supplemental Figures**


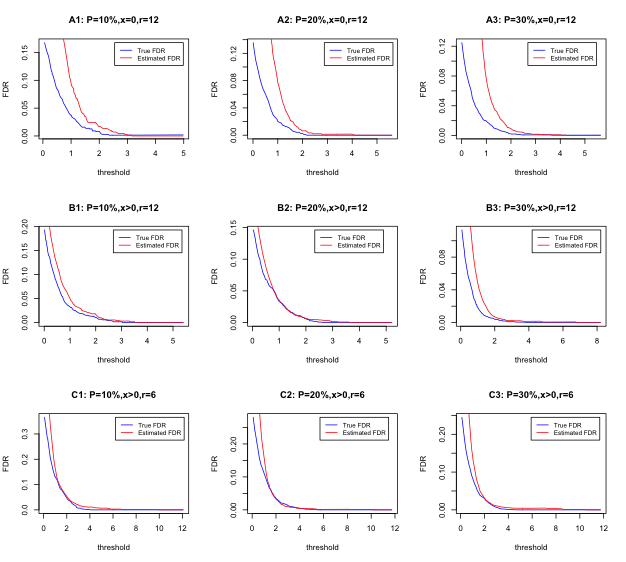


Figure S1

**Figure S1: Estimation of the False Discovery Rate.**

**(A)** False Discovery Rate (FDR) was estimated by using *x* = 0 across a set of given thresholds where the treated chi-squares and estimated null chi-squares were obtained from simulated data in which 16047 tags are scattered in 10160 genes. Two cell states (conditions) were set to correspond to resting and 48 hours after stimulation, 5 replicate libraries were set for cell states, and association effects were randomly assigned to 10% (in A1), 20% (in A2) and 30% (in A3) of poly(A) site states. The true value of FDR was computed by using the number of truly false positives detected divided by number of findings by RAX2. $r=\left(r_{1}+r_{2}\right)$ = 12. **(B)** FDR was estimated by using $x=\left(r/\chi_{S}^{2}(n)\right)^{2}$ across a set of given thresholds where $r=\left(r_{1}+r_{2}\right)$ = 12. The treated chi-squares and estimated null chi-squares were derived from the simulated data in (A). **(C)**: FDR was estimated using$x=\left(r/\chi_{S}^{2}(n)\right)^{2}$ across a set of given thresholds using simulated data with 18155 tags scattered in 10810 genes. The association effects were randomly assigned to 10% (in C1), 20% (in C2), and 30% (in C3) and 3 replicate libraries were set for each cell state$\left(r_{1}=r_{2}=3\right)$ and $r=\left(r_{1}+r_{2}\right)$= 6.


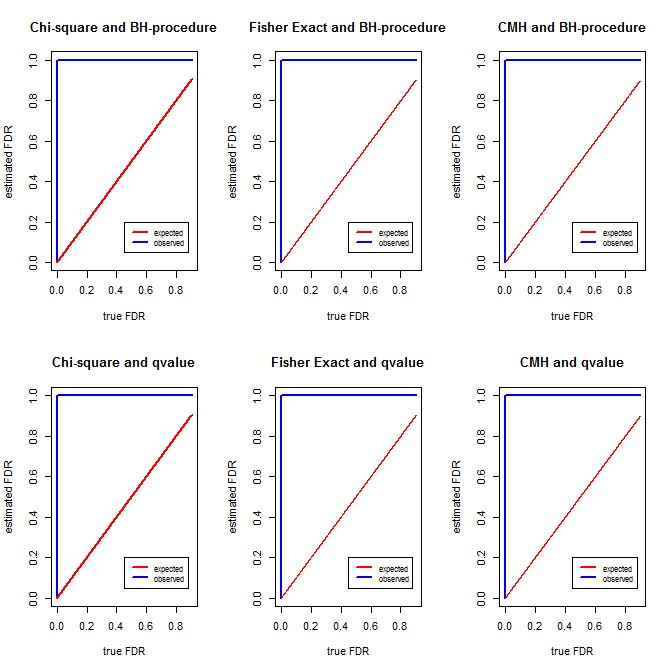


Figure S2

Figure S2: **Plots of estimated FDR versus true FDR in Person Chi-square, Fisher Exact test and CMH Chi-square test findings**

Expected FDR curve is that curve is given by estimated FDR equal to true FDR across all cutoff points. Benjamini-Hochberg and qvalue procedures were used to estimate FDR in positive tags found by Person chi-square test, Fisher exact tests and Cochran Mantel Haenszel (CMH) chi-square test in simulated datasets containing10% and 3 replicate libraries.


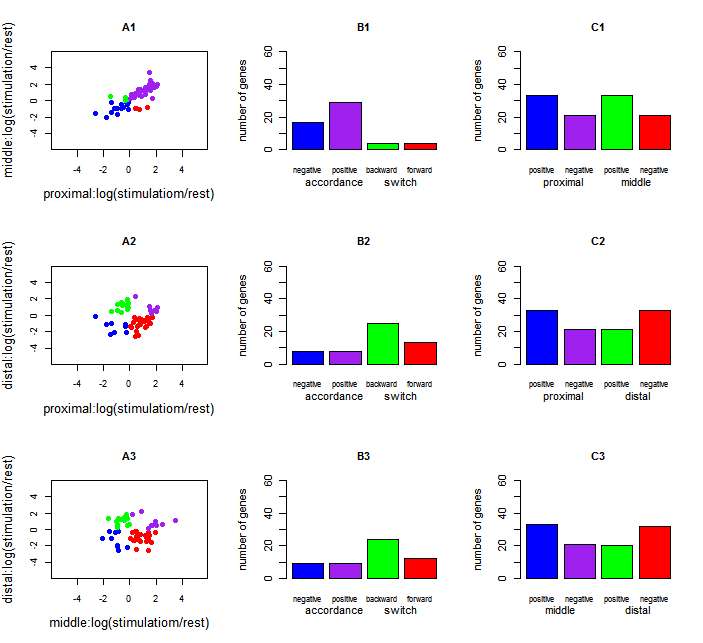


**Figure S3.** **Three** t**wo-way scatter plots for four association patterns of transcription of three tags within genes between two cell states**

Three two-way scatter plots display distributions of scatter dots in four phases: Phase II for forward switch (green dots), phase V for backward switch (red dots), phase I for positive accordance (purple dots) and phase III for negative accordance (blue dots). Numbers of genes in the four phases are given by histogram. Coordinates *y* and *x* are difference between ratios of counts of tags in stimulation and those in rest state. A1: proximal tags versus middle tags. A2: middle tags versus distal tags and A3: proximal tags versus distal tags. B1-B3: numbers of genes with accordantly positive and negative tags and forward and backward switching tags in A1-A3; C1: numbers of genes with proximal and middle tags positively or negatively response to stimulation; C2: numbers of genes with proximal and distal tags positively or negatively response to stimulation; C3: numbers of genes with middle and distal tags positively or negatively response to stimulation.

**User Guidance for RAX2**

RAX2 works especially on transcriptomic data derived replicate libraries. It is the first R package to perform large-scale analysis of associations between transcription variation of isoforms within genes and condition change across genome. RAX2 is a ranking analysis of chi-squares, take comparison between a set of ranked treatment chi-square statistics and a set of ranked null chi-square values across a set of given thresholds and estimation of a false discovery rate (FDR) profile.

1. **Getting RAX2**

You can download RAX2 package from Bioconductor or supplemental package.

1. **Installation of RAX2**

In current version downloaded from Supplemental package, RAX2 is a fold, not yet a package. So you open fold and load 5 R files into R Console using source/load, for example

source("C:/RAX2_pckg/RAX2_pckg/MultiChisquare_simulat.R")

source("C:/RAX2_pckg/RAX2_pckg/simulatX2.R")

source("C:/RAX2_pckg/RAX2_pckg/MultiChisquare2.R")

source("C:/RAX2_pckg/RAX2_pckg/RankingChisquare.R")

source("C:/RAX2_pckg/RAX2_pckg/simulatX2.R")

source("C:/RAX2_pckg/RAX2_pckg/chisqNormalization.R")

source("C:/RAX2_pckg/RAX2_pckg/subdata.R")

source("C:/RAX2_pckg/RAX2_pckg/twowayScatt.R")

source("C:/RAX2_pckg/RAX2_pckg/threeSitesTwowayScatt.R")

You need change directory in your computer or you can use setwd to set director in R Console, for example, here ("/Users/ C:/RAX2_pckg/RAX2_pckg /RAX2/”) is RAX2 in computer directory, so in R Console I set up setwd("C:/RAX2_pckg/RAX2_pckg /RAX2/”). Then using Source to input RAX2 file can remove “C:/RAX2_pckg/RAX2_pckg”.

.

1. **Import data into R Console**

Using read.csv( ) loads your data into R Console. For example,

ntcell<-read.csv("C:/RAX2_pckg/RAX2_pckg/RAX2/newt_tagflt_nosg_06182012.csv").

If you set up setwd, then you can directly load data into R Console, for example:

ntcell<-read.csv("newt_tagflt_nosg_06182012.csv")

The data frame, for example, in newt_tagflt_nosg_06182012.csv file is

> ntcell[1:4,]

tagid geneid name chr strand pos anno ST1.R ST3.R ST4.R ST5.R ST2.R ST1.S ST2.S ST3.S ST4.S ST5.S

1 608629 30216 B3GALT6 chr1 + 1169105 tu 9.2 5.8 1.7 3.2 2.1 5.7 1.7 4.2 3.5 4.6

2 352287 30216 B3GALT6 chr1 + 1170423 tu 3.7 11.5 7.3 4.7 1.0 23.2 3.9 26.8 10.1 25.3

3 4426604 30224 CPSF3L chr1 - 1246979 me-ce 22.6 18.1 14.2 16.5 41.4 39.5 6.2 16.1 37.7 66.9

4 861216 30224 CPSF3L chr1 - 1254061 me-ce 1.9 2.0 1.0 1.0 1.0 5.0 1.0 1.0 1.8 10.8

Your data must have at least geneid column and count columns. In the above data, geneid, tagid, gene name, chromosome, strand, position and poly(A) site types are listed in columns 1-7. This dataset has two conditions: rest(R) and stimulation(S). Each condition has 5 replicate libraries. You must normalize your count data so that all libraries have the same size.

1. **Perform RAX2**

Step 1: using mX2(xx,r1,r2*,* ns) to perform RAX2 analysis of your data. Here

xx is your transcriptomic data;

r1 is number of replicates in condition 1

r2 is number of replicates in condition 2

ns is iteration number. You can choose 10

In our example, ntcell is data consisting of 5 replicates and two conditions, so we have

ntmX2<-mX2(xx=ntcell,r1=5,r2=5, ns=10)

When run is ended, it would show a scatter plot, for example,


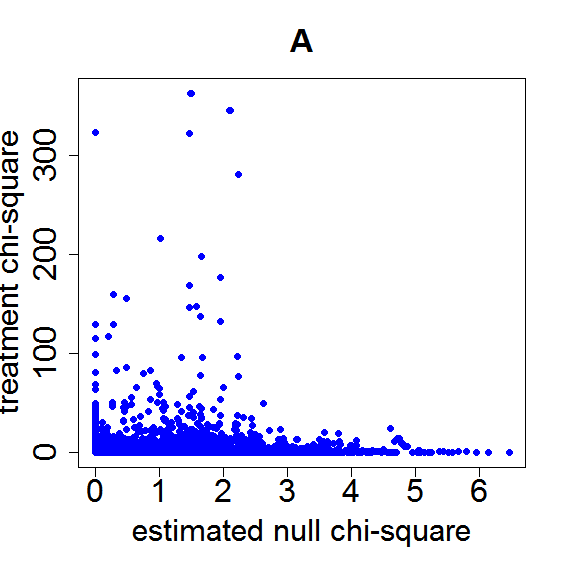


Step 2: using rankX2(yy,mX2,dn,r1,r2,alpha, file1,file2,file3)to do ranking analysis of chi-squares across transcriptome.

yy is data,

mX2 is a set of multiple chi-square values from step 1.

dn is number of deltas (thresholds),

r1 is number of replicates in condition 1

r2 is number of replicates in condition 2

alpha is significant level of test, you may choose 0.05 or 0.01.

file1 saves isoform information such as isoform id, geneid, gene name, chromosome id, strand, position, annotation, X2t, X2n, BH-adjusted alpha, number of isoforms.

File2 saves original but reordered data.

File 3 saves test results including estimated FDR and number of isoforms:

Example:

rankX2(yy= ntcell,mX2=ntmX2,dn=20,r1=5, r2=5,alpha=0.05,

file1="C:/RAX2/ Rnew_tcell_1PA.csv",

file2="C:/RAX2/ RX2_new_tcell_1PA. csv",

file3="C:/RAX2/ new_tcell_1PA_X2_result.csv")

And then you will get a linear plot, for example,


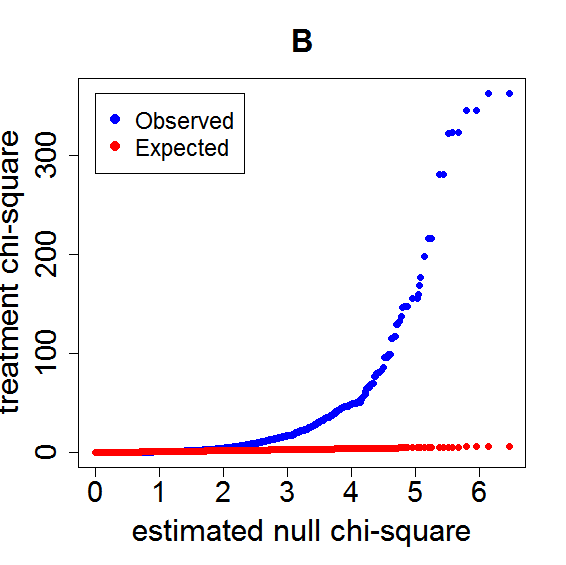


From File 3, you can determine number of isoforms associated with change in condition by choosing estimated FDR, for example, FDR<0.05 or FDR<0.01.

Figures 1 and 2 have high quantity for publication. Their zooms can be reduced very small, they still clear.

When you find number of associated isoforms, you can open file2 and find number of associated isoforms at last column. From that number, you go down to 1, you get your isoforms associated with change in condition in transcription process.

If you want to find two isoforms (tags) within genes, you copy the data from file 2 with these tags detected to be associated with condition to new excel file, and save the file as csv format. Then you can use subdata function to do that.

1. **Switch and accordance analysis**
2. **Two sites:**

subdata(xx,s,file)

xx is data from file2, s is number of poly(A) or splice site within genes that you want to choose, here s=2. file is output file with csv format.

For example, we choose 1610 tags at FDR<0.05 level and open the file “C:/RAX2/ RX2_new_tcell_1PA.csv” and copy the data with these 1610 tags(see the following figure)


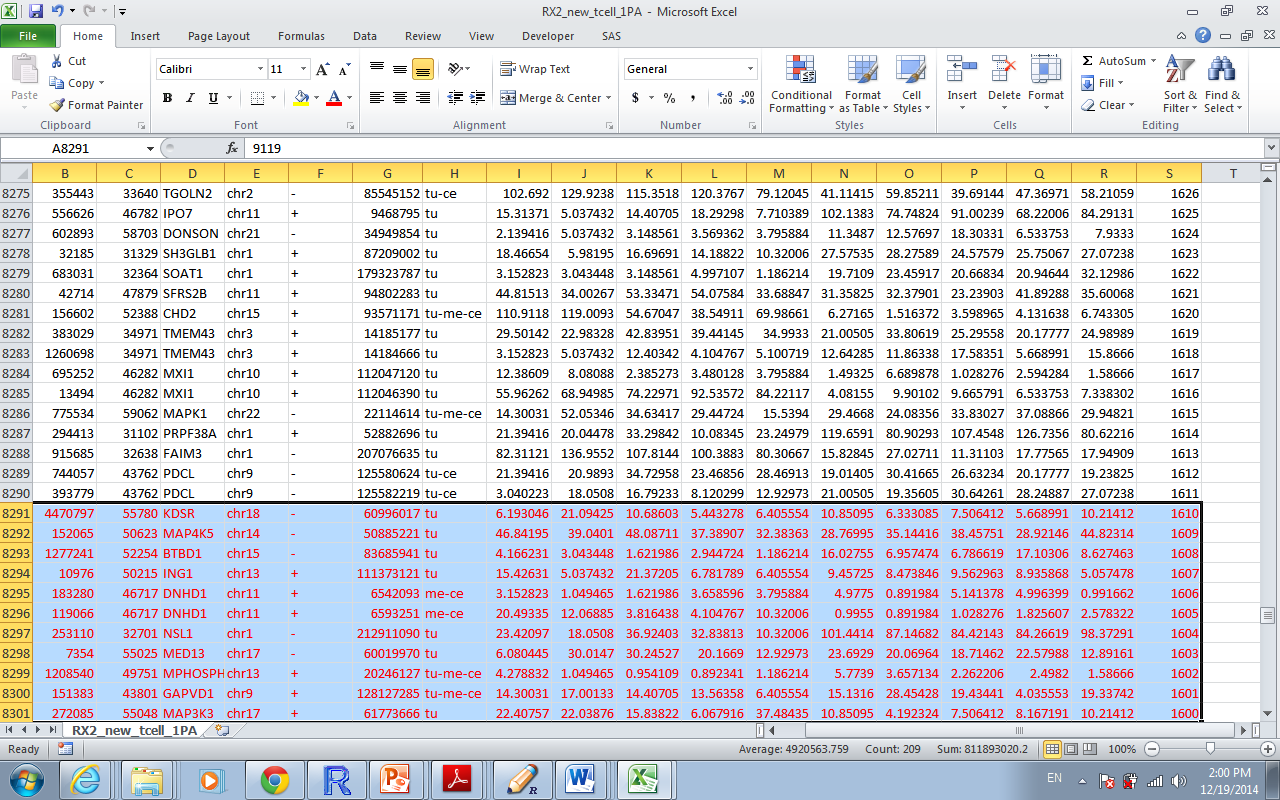


to new excel file and save it as “1610associated_tags_data.csv” in fold RAX2_revised version 6 in C driver. We then imported the file into R Console using read.csv:

>tags<-read.csv("C:/RAX2_revised version 6/1610associated_tags_data.csv")

We used subdata function to choose genes with two tags paired:

>subdata(xx=tags, s=2, file="C:/RAX2_revised version 6/associateed_two_PAsites_tag_data.csv").

There is a function twowayScatt in RAX2 package. You first open the csv file of genes with two-tag data created by subdata, delete the first column (order number change) and last column gg (tag number) and save it in your fold in a hard drive. You import this file into R Console. Here is an example:

>tag<-read.csv("C:/RAX2_revised version 6/associateed_two_PAsites_tag_data1.csv")

> tag[1:10,]

tagid geneid name chr strand pos anno ST1.R ST3.R

1 976871 30261 GNB1 chr1 - 1716730 tu 115.866262 135.905717

2 1369351 30261 GNB1 chr1 - 1718192 tu 34.681058 20.044781

3 179377 30319 RPL22 chr1 - 6246274 tu 527.309722 401.630255

4 252224 30319 RPL22 chr1 - 6246671 tu 1124.544563 1370.601288

5 374934 30344 VAMP3 chr1 + 7841489 tu 84.450628 107.989948

6 564441 30344 VAMP3 chr1 + 7839839 tu 3.152823 3.043448

7 452276 30389 TARDBP chr1 + 11085547 tu 9.233269 22.038765

8 650636 30389 TARDBP chr1 + 11084100 tu 56.976024 62.967900

9 412966 30421 TNFRSF8 chr1 + 12203095 tu 1.126008 1.049465

10 452298 30421 TNFRSF8 chr1 + 12204268 tu 2.139416 1.049465

ST4.R ST5.R ST2.R ST1.S ST2.S ST3.S

1 115.2564135 130.2817266 114.113759 107.11580 151.458848 135.012584

2 45.1293738 36.7644324 24.554624 76.15575 61.100890 66.632258

3 368.6678657 316.6024425 484.924165 120.75415 191.954913 147.454719

4 1316.2893050 1461.1184870 1448.604183 1080.01794 959.060977 1042.260134

5 53.2393036 67.1932467 75.087328 23.09560 18.642461 21.388132

6 2.3852735 3.5693624 2.491049 0.99550 7.314267 1.542413

7 18.2234892 12.1358321 18.030448 10.75140 13.290559 8.123377

8 68.2188209 83.9692498 60.852763 172.71925 142.539010 200.102429

9 0.9541094 0.8923406 2.491049 4.97750 1.516372 3.598965

10 0.9541094 1.6062131 1.186214 54.85205 24.707951 36.400956

ST4.S ST5.S

1 129.906377 145.675212

2 85.130954 78.638831

3 155.560965 132.981933

4 862.935780 876.332097

5 22.579881 13.585775

6 5.861161 1.586660

7 11.434067 5.751642

8 151.525412 161.938476

9 4.996399 3.768317

10 47.465792 44.029812

You can see that in this tag file, geneids are paired. Then you use twowayscatt function to perform switch and accordance analysis of genes with two tags(proximal and distal tags):

twowayscatt(xx,r1,r2,method,scale)

xx is data of genes with two tags or isoforms as seen above.

r1 and r2 are respectively sample sizes in conditions 1 and 2.

method is a method to select plot or barplots. method= “plot” will produce two way scatter plots seen in Figure 6A where proximal log(stimulation/rest) versus distal log(stimulation/rest) displays four patterns of tag or isoform transcription or splicing. method= “barplot” will produce histogram, as seen in Figure 6B, which shows numbers of genes with negative and positive tags and forward and backward switches corresponding to Figure 6A. method=”barplot1” will produce a histogram, as seen in Figure C, which shows numbers of genes with proximal and distal tags or isoforms response to change in condition. scale is option for choosing scale of y-axis in histograms. Our example is

>twowayscatt(xx=tag,r1=5,r2=5,method="barplot1",scale=400)


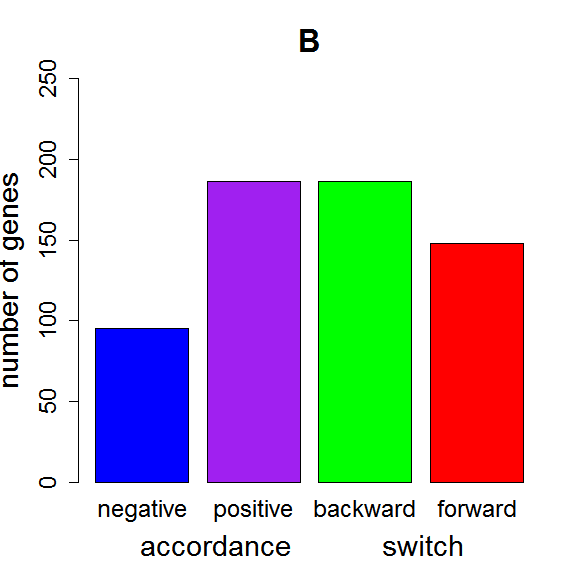


1. **Three sites:**

Like two-site switch and accordance analysis, you need to import data that contain tags or isoforms that were detected to be associated with change in condition into R Console. Our current example is

>tags<-read.csv("C:/RAX2_revised version 6/1610associated_tags_data.csv")

tags are data containing 1610 tags of being detected by RAX2 to be associated with stimulation. Then

using subdata(xx,s,file) and set s=3, you can create a file of genes with only three tags or isoforms detected to be associated with change in condition from the data you imported into R Console. So for our current example, we have

> subdata(xx=tags,s=3,file=” associateed_three_PAsites_tag_data.csv”)

Then we created a file named as associateed_three_PAsites_tag_data.csv. We opened the file and deleted the first and last column and saved it:


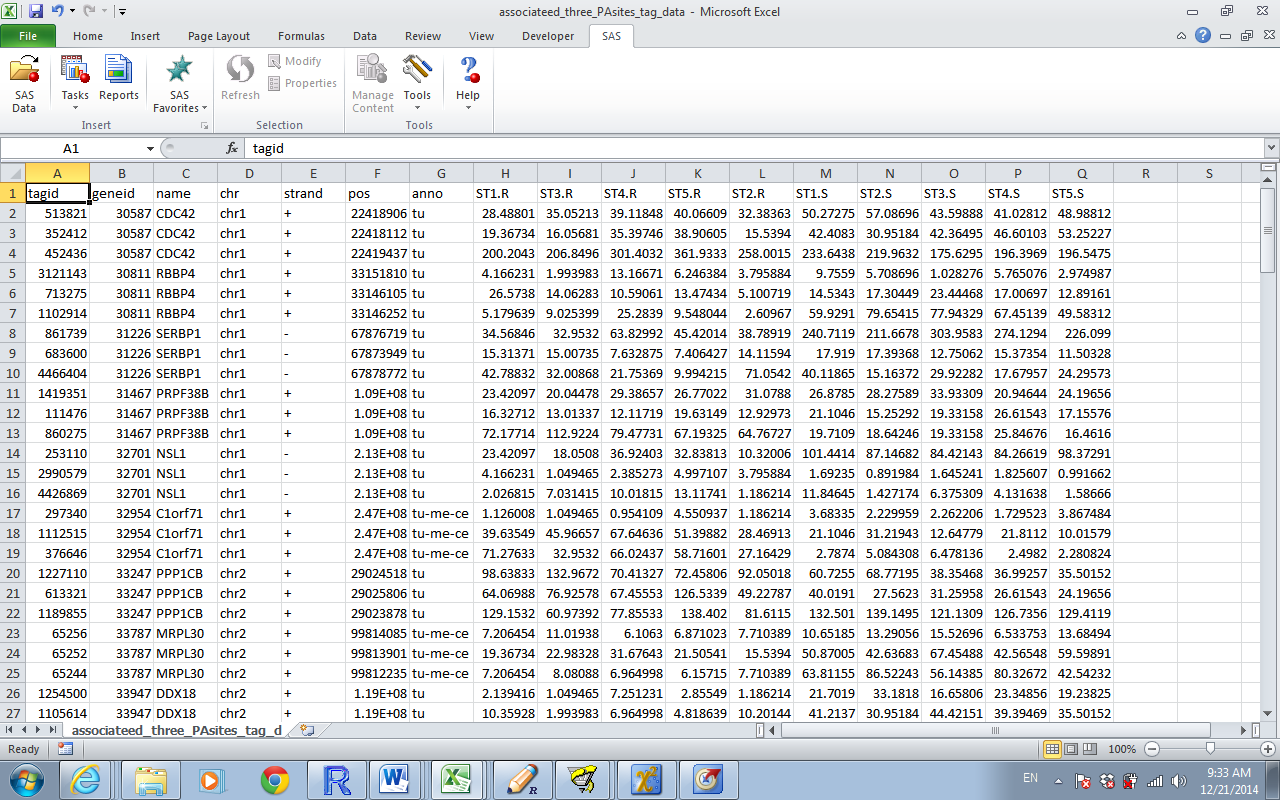


From the csv file, you can see that geneids and names are triplicates.

Import the file into R Console by using read.csv():

> tag3<-read.csv("C:/RAX2_revised version 6/associateed_three_PAsites_tag_data.csv")

> tag3[1:3,]

tagid geneid name chr strand pos anno ST1.R ST3.R ST4.R

1 513821 30587 CDC42 chr1 + 22418906 tu 28.48801 35.05213 39.11848

2 352412 30587 CDC42 chr1 + 22418112 tu 19.36734 16.05681 35.39746

3 452436 30587 CDC42 chr1 + 22419437 tu 200.20429 206.84955 301.40315

ST5.R ST2.R ST1.S ST2.S ST3.S ST4.S ST5.S

1 40.06609 32.38363 50.27275 57.08696 43.59888 41.02812 48.98812

2 38.90605 15.53940 42.40830 30.95184 42.36495 46.60103 53.25227

3 361.93334 258.00148 233.64385 219.96320 175.62947 196.39692 196.54750

Then you can use function twowayscatt3(xx,r1,r2,scale) to create a 3×3 plot where xx is data that imported into R Console, r1 and r2 are sample sizes or replicate numbers in conditions 1 and 2, respectively, and scale is scale value of y-axis in histograms. Our current example is

> twowayscatt3(xx=tag3,r1=5,r2=5,scale=60)

You try several times and determine the best scale value. You will create figure as seen in Figure S3. This figure displays three scatter plots and histograms of proximal tags versus middle tags, proximal tags against distal tags and middle tags against distal tags.
