# Supplementary material for: RAX2: a genome-wide detection method of condition-associated transcription variation
Source: Nucleic Acids Res. 2015 May 7;43(15):e96. doi: 10.1093/nar/gkv411 (PMC4551904; doi:10.1093/nar/gkv411)
Supplement: SUPPLEMENTARY DATA [file supp_gkv411_nar-00147-met-n-2015-File013.zip › RAX2 User Guideline.docx]

RAX2 User Guideline

Overall

RAX2 works specially on transcriptomic data. This is so far the first R program to perform large-scale tests for association between transcription variation of isoforms within genes and conditional change. Current version is called RAX2I because just one isoform within genes is considered. In the updated version, we will have RAX2II in two conditions and in multiple conditions. RAX2II will perform chi-square test for association between transcription variation of two isoforms within each gene and change in condition across genome.

Getting RAX2

You need email to Yuande Tan or Jeol R. Neilson for requesting RAX2. Yuande Tan’s email: [tanyuande@gmail.com](mailto:tanyuande@gmail.com), Jeol R. Neilson’s email: [eilson@bcm.edu](mailto:eilson@bcm.edu)

Installation of RAX2

In current version, RAX2 is a fold, not a package. So you open fold and load 5 R files into R workspace using Source/Load, for example

> source("/Users/ytan/RAX2/MultiChisquare_simulat.R")

> source("/Users/ytan/RAX2/simulatX2.R")

> source("/Users/ytan/RAX2/MultiChisquare2.R")

> source("/Users/ytan/RAX2/RankingChisquare.R")

> source("/Users/ytan/RAX2/simulatX2.R")

> source("/Users/ytan/RAX2/chisqNormalization.R")

> source("/Users/ytan/RAX2/subdata.R")

You need change directory in your computer.

Import data into R workspace

Using read.csv( ) loads your data into R workspace. For example,

ntcell<-read.csv("/Users/ytan/RAX2/newt_tagflt_nosg_06182012.csv")

look at your data, for example, our data in 1 to 4 rows are

> ntcell[1:4,]

tagid geneid name chr strand pos anno ST1.R ST3.R ST4.R ST5.R ST2.R ST1.S ST2.S ST3.S ST4.S ST5.S

1 608629 30216 B3GALT6 chr1 + 1169105 tu 9.2 5.8 1.7 3.2 2.1 5.7 1.7 4.2 3.5 4.6

2 352287 30216 B3GALT6 chr1 + 1170423 tu 3.7 11.5 7.3 4.7 1.0 23.2 3.9 26.8 10.1 25.3

3 4426604 30224 CPSF3L chr1 - 1246979 me-ce 22.6 18.1 14.2 16.5 41.4 39.5 6.2 16.1 37.7 66.9

4 861216 30224 CPSF3L chr1 - 1254061 me-ce 1.9 2.0 1.0 1.0 1.0 5.0 1.0 1.0 1.8 10.8

>

Perform RAX2

Step 1: using smmX2(xx,dn,r1,r2,p,a,K,ns) to simulate a and K values.

xx is transcriptomic data, dn=200 is not used,r1 and r2 are replicate numbers of conditions 1 and 2, respectively=0 for null simulation, a is theta value in our paper, a and K are parameters for estimating null chi-square distribution. ns is iteration number or simulation times.

First all, you can set a=1,K=1 and ns=2 and look at chi-square distribution:

smmX2(xx=ntcell, dn=200, r1=5,r2=5,p=0,a=1,K=1,ns=2)

The result shows as follows


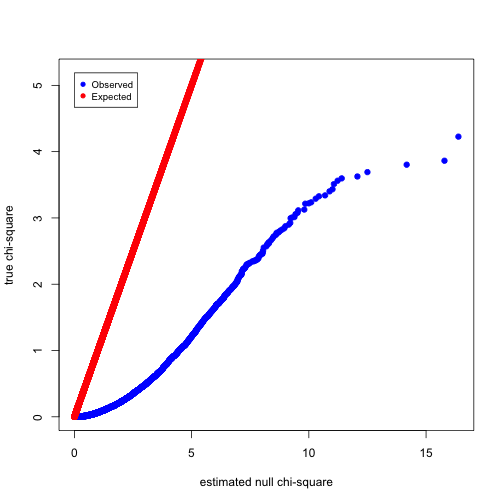


Figure 1

Figure1 shows that null chi-square distribution is overestimated, and observed curve is not linear, so it needs to adjust parameters *a* and K. Here we tried to set a=5 and K=3.3:

smmX2(xx=ntcell, dn=200, r1=5,r2=5,p=0,*a*=5,K=3.3,ns=2)

The result is shown in Figure 2. From Figure 2, we see that estimated null chi-square distribution is almost identical to true null chi-square distribution.
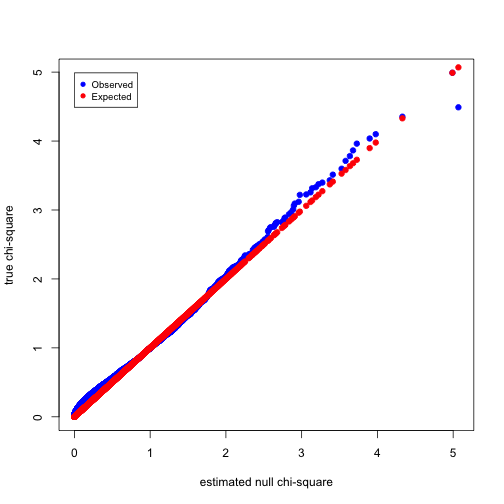


Figure 2

Then we setup ns=20 to get smooth and linear observed chi-square curve overlapped with expected curve:

smmX2(xx=ntcell, dn=200, r1=5,r2=5,p=0,*a*=5,K=3.3,ns=20)


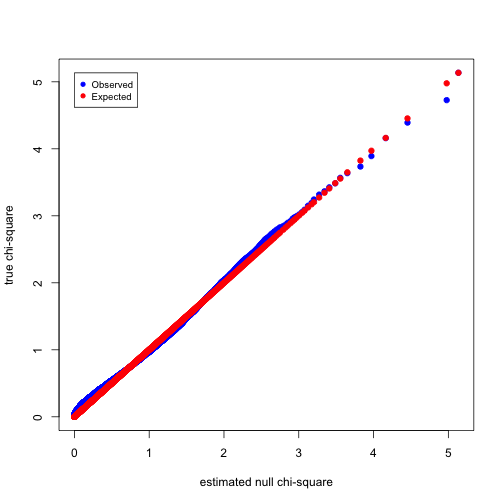


Figure 3

Figure 3 shows that observed and expected curves almost overlap, therefore, estimated null chi-square distribution is true null chi-square distribution.

Step 2: using mX2(xx,r1,r2*,a*,K,ns)to perform RAX2 analysis of your data. Here

xx is your transcriptomic data;

r1 is number of replicates in condition 1

r2 is number of replicates in condition 2

a and K have been determined by simulation. In this data, *a*=5, K=3.3

ntmX2<-mX2(xx=ntcell,r1=5,r2=5, *a*=5,K=3.3,ns=20)

After finishing running, it shows a scatter plot, for example,


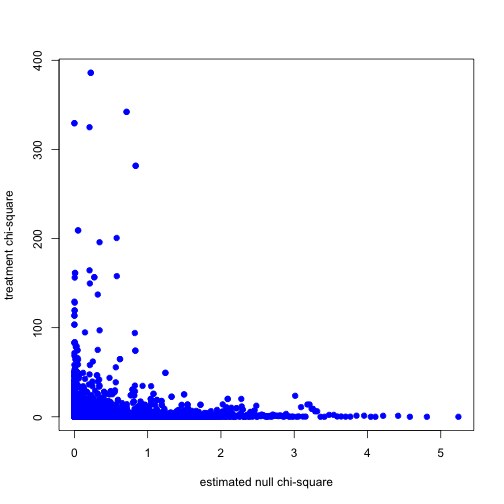


Figure 4

Step 3: using rankX2(yy,mX2,dn,r1,r2,alpha, file1,file2,file3)to do ranking analysis of chi-squares across all transcriptome.

yy is your data,

mX2 is a set of multiple ch-square values from step 2.

dn is number of deltas (thresholds),

r1 is number of replicates in condition 1

r2 is number of replicates in condition 2

alpha is significant level of test,

file1 saves isoform information such as isoform id, geneid, gene name, chromosome id, strand, position, annotation, X2t, X2n, BH-adjusted alpha, number of isoforms.

File2 save original data.

File 3 save test results including estimated FDR and number of isoforms:

rankX2(yy=ntcell,mX2=ntmX2,dn=200,r1=5,r2=5,alpha=0.05, file1=” Rnew_tcell_1PA.csv”,file2=” RX2_new_tcell_1PA.csv”,file3=” new_tcell_1PA_X2_result.csv”)

You will get a linear plot, for example,
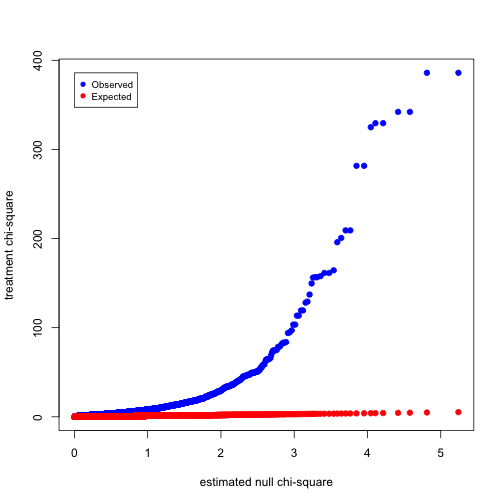


Figure 5

and three files with .csv.

From File 3, you can determine number of isoforms associated with conditional change by choosing estimated FDR, for example, FDR<0.05 or FDR<0.01.

When you find number of associated isoforms, you can open file2, find number of associated isoforms at last column. From that number to 1, you get your isoforms within genes associated with conditional change in transcription process.

If you want to find two isoforms within genes, you can use subdata function to do that.

subdata(xx,file)

xx is your data or file2 from RAX2.

File is output file.
